# Lysophosphatidic acid receptor 1 (LPA_1_) plays critical roles in microglial activation and brain damage after transient focal cerebral ischemia

**DOI:** 10.1186/s12974-019-1555-8

**Published:** 2019-08-20

**Authors:** Bhakta Prasad Gaire, Arjun Sapkota, Mi-Ryoung Song, Ji Woong Choi

**Affiliations:** 1 Laboratory of Neuropharmacology, College of Pharmacy and Gachon Institute of Pharmaceutical Sciences, Gachon University, Yeonsu-gu, Incheon, 406-799 Republic of Korea; 2 School of Life Sciences, Gwangju Institute of Science and Technology, Buk-gu, Gwangju, 500-712 Republic of Korea

**Keywords:** Lysophosphatidic acid receptor 1, AM095, shLPA_1_, Middle cerebral artery occlusion/reperfusion, Microglial activation, Proinflammatory cytokines

## Abstract

**Background:**

Lysophosphatidic acid receptor 1 (LPA_1_) is in the spotlight because its synthetic antagonist has been under clinical trials for lung fibrosis and psoriasis. Targeting LPA_1_ might also be a therapeutic strategy for cerebral ischemia because LPA_1_ triggers microglial activation, a core pathogenesis in cerebral ischemia. Here, we addressed this possibility using a mouse model of transient middle cerebral artery occlusion (tMCAO).

**Methods:**

To address the role of LPA_1_ in the ischemic brain damage, we used AM095, a selective LPA_1_ antagonist, as a pharmacological tool and lentivirus bearing a specific LPA_1_ shRNA as a genetic tool. Brain injury after tMCAO challenge was accessed by determining brain infarction and neurological deficit score. Role of LPA_1_ in tMCAO-induced microglial activation was ascertained by immunohistochemical analysis. Proinflammatory responses in the ischemic brain were determined by qRT-PCR and immunohistochemical analyses, which were validated in vitro using mouse primary microglia. Activation of MAPKs and PI3K/Akt was determined by Western blot analysis.

**Results:**

AM095 administration immediately after reperfusion attenuated brain damage such as brain infarction and neurological deficit at 1 day after tMCAO, which was reaffirmed by LPA_1_ shRNA lentivirus. AM095 administration also attenuated brain infarction and neurological deficit at 3 days after tMCAO. LPA_1_ antagonism attenuated microglial activation; it reduced numbers and soma size of activated microglia, reversed their morphology into less toxic one, and reduced microglial proliferation. Additionally, LPA_1_ antagonism reduced mRNA expression levels of proinflammatory cytokines and suppressed NF-κB activation, demonstrating its regulatory role of proinflammatory responses in the ischemic brain. Particularly, these LPA_1_-driven proinflammatory responses appeared to occur in activated microglia because NF-κB activation occurred mainly in activated microglia in the ischemic brain. Regulatory role of LPA_1_ in proinflammatory responses of microglia was further supported by in vitro findings using lipopolysaccharide-stimulated cultured microglia, showing that suppressing LPA_1_ activity reduced mRNA expression levels of proinflammatory cytokines. In the ischemic brain, LPA_1_ influenced PI3K/Akt and MAPKs; suppressing LPA_1_ activity decreased MAPK activation and increased Akt phosphorylation.

**Conclusion:**

This study demonstrates that LPA_1_ is a new etiological factor for cerebral ischemia, strongly indicating that its modulation can be a potential strategy to reduce ischemic brain damage.

**Electronic supplementary material:**

The online version of this article (10.1186/s12974-019-1555-8) contains supplementary material, which is available to authorized users.

## Introduction

Lysophosphatidic acid (LPA) is a bioactive lysophospholipid found in extracellular compartment such as blood and cerebrospinal fluid [1]. LPA functions through binding to its specific six G protein-coupled receptors (LPA_1–6_) that are expressed on a variety of cell types and control cellular functions specifically by activating diverse effector pathways [2]. Many studies utilizing genetic null mutants or chemical modulators have revealed that receptor-mediated LPA signaling is a critical pathogenic factor for diverse diseases, which indicates LPA receptors as considerable therapeutic targets [2–4]. Up to date, most pharmacological advances have focused on the modulation of LPA_1_, the first identified LPA receptor subtype, in diverse disease types such as fibrosis, systemic sclerosis, and cancer [4]. In particular, a selective antagonist of LPA_1_ is under several clinical trials for the treatment of lung fibrosis (ClinicalTrials.gov ID: NCT01766817) and psoriasis (ClinicalTrials.gov ID: NCT02763969) [4]. In the nervous system, emerging evidence indicates that LPA_1_ may become a considerable target to treat neurological diseases, including neuropathic pain, spinal cord injury, traumatic brain injury, neuropsychiatric disorders, hypoxia, and hydrocephalus [3–6].

In addition to these types of neurological diseases, cerebral ischemia can be a complementary disease type in which LPA_1_ may play a pivotal role in brain damage. Cerebral ischemia results from a sudden interruption of blood flow in the brain, leading to irreversible neural cell death [7]. One of diverse pathogenic mechanisms underlying neural cell death in acute and chronic phases of cerebral ischemia is inflammation that results in the production of proinflammatory cytokines, expression of adhesion molecules, and infiltration of immune cells into the ischemic brain [8–11]. Brain resident microglia are one of main cell types for this inflammation. Their activation is a critical event to control immune responses in the ischemic brain [12–14]. The importance of LPA_1_ in microglial activation in other disease types has been recently reported, in which LPA_1_ triggers microglial activation as a possible factor for secondary damage in spinal cord injury [6] and microglial activation and their TNF-α production in activated microglia by enhancing ERK1/2 phosphorylation in lipopolysaccharide (LPS)-induced septic brain [15]. These two independent studies clearly demonstrate that LPA_1_ influences microglial activation, indirectly implicating its pathogenic role in cerebral ischemia because microglial activation is a core pathogenic event of cerebral ischemia [16]. Intriguingly, a genetic deletion of LPA_1_ abolished neuropathic pain responses in mice challenged with a slight occlusion (15 min of occlusion)-induced transient focal cerebral ischemia [17] and photothrombotic ischemia [18]. However, whether LPA_1_ contributes to direct brain damage, such as brain infarction, neurological functional deficit, and neural cell death, and brain damage-relevant immune responses through microglial activation is currently unclear. Therefore, it is tempting to determine whether LPA_1_ can be a pathogenic factor for direct brain injury in cerebral ischemia.

In the present study, we determined the role of LPA_1_ in a transient focal cerebral ischemia that was induced by 90 min of middle cerebral artery occlusion (MCAO) and reperfusion (tMCAO) combined with pharmacological and genetic approaches to suppress LPA_1_ activity. We report that LPA_1_ contributes to brain damage after tMCAO challenge because suppressing LPA_1_ activity reduced brain infarction and improved neurological function. Importantly, we report that the role of LPA_1_ may be associated with microglial activation, morphological transition to more toxic cells, proliferation, and proinflammatory responses in the ischemic brain.

## Materials and methods

### Animals

Male ICR mice (32 ± 2 g, 6 weeks old) were purchased from Orient Co., Ltd. (Gyeonggi-do, Korea) and housed in controlled conditions (temperature (22 ± 2 °C), relative humidity (60 ± 10), and a 12-h light/dark cycle (light on 07.00–19.00)). They were provided free access to food and water ad libitum. Mice were allowed to acclimatize in the laboratory environment for a week. All mice handling and surgical procedures were performed in compliance with the Institutional Animal Care and Use guidelines of Lee Gil Ya Cancer and Diabetes Institute (LCDI) at Gachon University (animal protocols approved number: LCDI-2013-0074, LCDI-2014-0079, LCDI-2018-0051, and LCDI-2019-0027). Scheme of experimental procedure was shown in Additional file 1: Figure S1.

### Induction of transient focal cerebral ischemia in mice

Transient focal cerebral ischemia was induced by MCAO and reperfusion (tMCAO) using an intraluminal suture method as described previously [19, 20]. Briefly, mice were anesthetized with isoflurane (3% for induction and 1.5% for maintenance) in N_2_O∶O_2_ (3∶1) and fixed in an operating frame. After separating the right common carotid artery (CCA) from the vagus nerve, MCAO was induced by inserting a 9-mm-long 5–0 nylon monofilament coated with silicon from CCA bifurcation to the MCA. After 90 min of ischemic period, blood flow was restored by withdrawing the monofilament to allow complete reperfusion of the ischemic area under anesthesia. Body temperature of mice was maintained at 37 ± 0.5 °C during surgery. Sham-operated mice underwent the same surgical procedure without occlusion of MCA.

### AM095 administration

First, tMCAO-challenged mice were randomly divided into vehicle (1% DMSO in 10% Tween-80)- or AM095-administered group. AM095 (30 mg/kg) was administered to mice by oral gavage using a mouse feeding tube immediately after reperfusion to determine its therapeutic effect on brain damage caused by cerebral ischemia. Alternatively, vehicle or AM095 was administered to mice 1 h prior to tMCAO surgery to determine its prophylactic effects in the ischemic brain. The dose of AM095 was chosen based on previous reports [6, 21, 22]. The investigator was blinded to the treatment groups.

### Intracerebroventricular injection of LPA_1_ shRNA lentivirus particles

Stereotaxic injection of lentivirus particle was done as described previously [20]. Briefly, mice were anesthetized by intramuscular injection of Zoletil 50® (mixture of tiletamine and zolazepam, 10 mg/kg) and Rompun® (xylazine, 3 mg/kg) mixture. Mouse head was fixed with stereotaxic apparatus. LPA_1_ shRNA (shLPA_1_, MISSION® lentiviral transduction particles, sequence: CCGGTGTTCAATACAGGACCTAATACTCGAGTATTAGGTCCTGTATTGAACATTTTTG, GenBank RefSeq: NM_010336, Sigma-Aldrich (St. Louis, MO, USA)) and non-target control shRNA (shNC, MISSION® TRC2 [SHC216V], Sigma-Aldrich) lentivirus particles (3 μl) were injected into the right lateral ventricle at 0.5 μl/min based on the mouse brain atlas with stereotaxic coordinates of 0.3 mm caudal to the bregma and 1.0 mm lateral to the midline at a depth of 3 mm [20]. After completing the injection, the syringe was withdrawn using an intermediate step with a 1 min inter-step delay to minimize backflow of lentivirus particles. Non-target control lentivirus (negative control) particles were injected in a similar manner. Mice were challenged with tMCAO 1 week after the infection. Knockdown efficiency of LPA_1_ shRNA lentivirus was confirmed using quantitative real-time PCR.

### Determination of functional neurological deficit score and infarct volume in the brain

Modified neurological severity score (mNSS) scale was used to access the motor and sensory function, reflex, and balance tests at 1 day or 3 days after tMCAO challenge. The sum of partial scores yielded total mNSS with a maximum of 18 points and minimum of 0 point in normal mice as described previously [19, 20].

After performing neurological deficit assessment, mice were sacrificed with CO_2_ inhalation. Their brains were quickly removed and cut into 2-mm-thick coronal sections. Brain slices were stained using 2% 2,3,5-triphenyltetrazolium chloride (TTC) in saline at 37 °C and photographed. The infarct volume of each slice was determined by dividing the infarct portion through the total volume of slice using ImageJ software (National Institute of Mental Health, Bethesda, MD, USA). Total infarct volume was calculated by summing partial infarct volume of each slice.

In total, nine mice died before the experimental end point. Among them, five mice were from vehicle-administered group, one mouse was from AM095-administered group, two mice were from shNC-infected group, and one mouse was from shLPA_1_-infected group. In addition, mice with cerebral hemorrhage (*n* = 6) were excluded from data analysis.

### Histological assessment

#### Tissue preparation

At 1 day or 3 days after ischemic challenge, mice were anesthetized using a mixture of Zoletil 50® (10 mg/kg, i.m.) and Rompun® (3 mg/kg, i.m.) and perfused transcardially with ice-cold phosphate-buffered saline (PBS, pH 7.4) followed by fixation with 4% paraformaldehyde (PFA). Removed brains were post-fixed in 4% PFA overnight, incubated with 30% sucrose solution, frozen in Tissue-Tek Optimal Cutting Temperature (OCT) compound, and cut into 20 μm sections using a cryostat (J4800AMNZ, Thermo, Germany).

#### Iba1 or GFAP immunohistochemistry

Cryostat brain sections were oxidized with 1% hydrogen peroxide (H_2_O_2_) in PBS for 15 min, blocked with 1% fetal bovine serum (FBS) in PBS containing 0.3% Triton X-100 for 1 h, and incubated with rabbit anti-Iba1 (1:500; Wako, Japan) or rabbit anti-GFAP (1:500; abcam, UK) antibody overnight at 4 °C. The sections were then labeled with secondary biotinylated antibody (1:200) followed by an ABC reagent (1:100, Vector Labs). Signals were visualized with DAB staining (0.02% DAB in 0.01% H_2_O_2_ for 5 min).

#### 5-Bromo-2′-deoxyuridine immunofluorescence

Microglia or astrocyte proliferation was evaluated using 5-bromo-2′-deoxyuridine (BrdU) immunofluorescence as described previously [20, 23]. Briefly, mice received 50 mg/kg BrdU (i.p., Sigma-Aldrich) twice per day for 2 days (12-h interval) after tMCAO or sham operation. First BrdU injection was made 12 h after reperfusion, and brain samples were obtained at 3 days after tMCAO challenge. Brain cryostat sections (20 μm) were incubated with pre-warmed 2 N HCl for 30 min at 37 °C, neutralized with 0.1 M borate buffer (pH 8.5) for three times (15 min each), and blocked with 1% FBS in 0.3% Triton X-100. These sections were incubated with primary antibodies against BrdU (1:200, abcam) and Iba1 or BrdU and GFAP overnight at 4 °C followed by incubation with secondary antibodies (1:1000) conjugated with AF488 (Invitrogen) and Cy3 (Jackson ImmunoResearch), counterstained with DAPI, and mounted with VECTASHIELD.

#### NF-κB immunofluorescence

The role of LPA_1_ in microglial NF-κB activation was determined by Iba1/NF-κB double immunofluorescence in post-ischemic brain at 3 days after tMCAO challenge. Brain sections were treated with TRIS-EDTA at 100 °C followed by blocking with 1% FBS and incubated with primary antibody against anti-Iba1 (1:500) and anti-NF-κB (1:200) overnight at 4 °C. These sections were then incubated with secondary antibodies (1:1000) conjugated with AF488 and Cy3 and mounted with VECTASHIELD.

#### Image preparation

Bright-field images were obtained with a microscope (BX53T, Olympus, Japan) equipped with a DP72 camera. Fluorescent images were obtained using a laser scanning confocal microscope (Eclipse A1 Plus, Nikon, Japan). Brain sections from 4~5 mice were analyzed for quantification of immunopositive cells. The number of immunopositive cells for each mouse was obtained by calculating the mean value from three images (20X) of each section as described previously [23]. Soma area was measured in each Iba1-immunopositive cell using ImageJ software, and soma size for each mouse was obtained by calculating the mean value. Amoeboid and ramified microglia were differentiated through their morphology as previously described [24, 25]. In brief, microglia with small soma and branched processes were designated as ramified microglia and those with larger round soma and reduced branched processes were designated as amoeboid microglia.

### Mouse primary microglial cell culture

Primary microglia were cultured from mouse cortices of 1- to 2-day-old mouse pups as described previously [20, 23]. Briefly, cerebral cortices were triturated into single cells, plated into T75 flasks, and cultured in DMEM containing 10% heat-inactivated FBS. After about 2 weeks, microglia were collected by gentle shaking, plated onto 12-well culture plate, and used for experiments. The purity of isolated microglia was > 95% as determined by an immunocytochemical analysis. Primary microglia were pretreated with AM095 for 30 min prior to LPS (100 ng/ml) exposure for additional 24 h.

### Transduction of primary microglia with LPA_1_ shRNA

Primary microglia were infected with lentivirus particles containing mouse LPA_1_-specific shRNA or non-target control shRNA in serum- and antibiotics-free DMEM containing 8 μg/ml polybrene. After 4 h, cells were cultured in DMEM containing 10% FBS and antibiotics for additional 72 h. Cells were then starved for 6 h and treated with LPS (100 ng/ml) for 24 h. Knockdown efficiency in infected cells with LPA_1_ shRNA lentivirus was confirmed using qRT-PCR.

### Quantitative real-time PCR

Total RNA was obtained from cells at the end of treatment or the ipsilateral brain hemisphere of mouse. Total RNA (1 μg) was reverse transcribed to cDNA which was then used as a template for qRT-PCR with FG Power SYBR® Green mix (Applied Biosystems) and primer sets (Additional file 10: Table S1) to detect specific PCR products using StepOnePlus™ qRT-PCR system (Applied Biosystems). The relative mRNA expression level of each target gene was calculated as fold change relative to control through the 2^-ΔΔCt^ method. β-actin, a housekeeping gene, was used as an internal control.

### Western blot analysis

Ischemic brain hemispheres were triturated using a neuronal protein extraction reagent (Thermo Fisher Scientific, USA). Proteins (30 μg) were subjected to SDS-PAGE gel electrophoresis, transferred on PVDF membranes, and blocked with 5% skim milk to prevent non-specific binding. These membranes were then incubated with primary antibodies against rabbit p-ERK1/2, ERK1/2, p-p38, p38, p-JNK, JNK, p-Akt, Akt (Cell signaling, 1:1000), and β-actin (Sigma Aldrich, 1:5000) at 4 °C overnight followed by incubation with horseradish peroxidase-conjugated secondary antibodies. Membranes were then developed with an enhanced chemiluminescence (ECL) detection kit (Thermo Fisher Scientific, USA). The ImageQuant (TM) TL software (GE Healthcare Bio-Science, Uppsala, Sweden) was used to quantify target protein bands. Protein expression levels were calculated as fold change relative to sham control after normalization against β-actin level.

### Statistical analysis

All data are presented as mean ± standard error of the mean (SEM). Statistical tests were performed using GraphPad Prism 5 (GraphPad Software Inc., La Jolla, CA, USA). Differences among groups were analyzed by a one-way analysis of variance (ANOVA) followed by the Newman-Keuls test for multiple comparisons whereas comparisons between two groups were performed using Student’s *t* test. Statistical significance was considered when *p* value was below 0.05.

## Results

### LPA_1_ is a critical factor for brain damage in mice challenged with transient focal cerebral ischemia

**Fig. 1:**
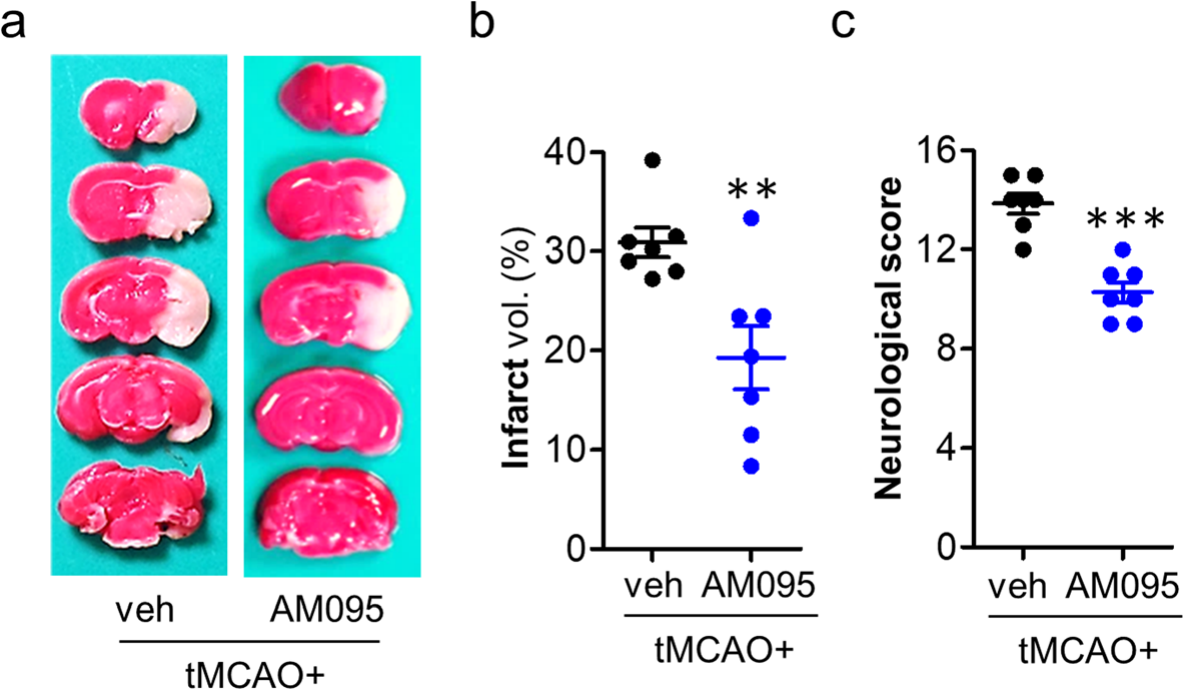
LPA_1_ antagonism reduces brain damage at 1 day after tMCAO-challenged. Mice were challenged with tMCAO. AM095 (30 mg/kg, p.o.) was then administered immediately after reperfusion. Brain damage was assessed at 1 day after tMCAO. **a**–**c** Effects of AM095 on infarct volume (**a**, **b**) and neurological function (**c**) were determined. Representative images of TTC-stained brain slices (**a**) and quantification of brain infarction (**b**). Neurological score indicating neurological functions (**c**). *n* = 7 mice per group. **p < 0.01 and ***p < 0.001 versus vehicle-administered tMCAO mice (tMCAO+veh)

To address whether LPA_1_ could mediate brain damage in cerebral ischemia, mice were challenged with tMCAO and received an LPA_1_ antagonist, AM095 (30 mg/kg, p.o.), immediately after reperfusion followed by assessment of brain damage at 1 day after tMCAO. Brain infarction was first assessed by TTC staining. In vehicle-treated tMCAO group, severe brain infarction was developed in both the cerebral cortex and striatum (30.99 ± 1.77%), which was markedly reduced by AM095 administration (19.15 ± 3.84%; Fig. 1a, b). Similarly, AM095 administration significantly improved neurological functions in ischemic mice compared with vehicle administration (Fig. 1c). In addition, AM095 administration 1 h prior to tMCAO challenge significantly prevented brain damage compared with vehicle administration as assessed by brain infarction (Additional file 2: Figure S2a, b) and neurological deficit score (Additional file 2: Figure S2c). These data demonstrate that pharmacological inhibition of LPA_1_ can reduce brain damage in tMCAO-challenged mice, clearly suggesting that LPA_1_ signaling contributes to brain damage in cerebral ischemia.

**Fig. 2:**
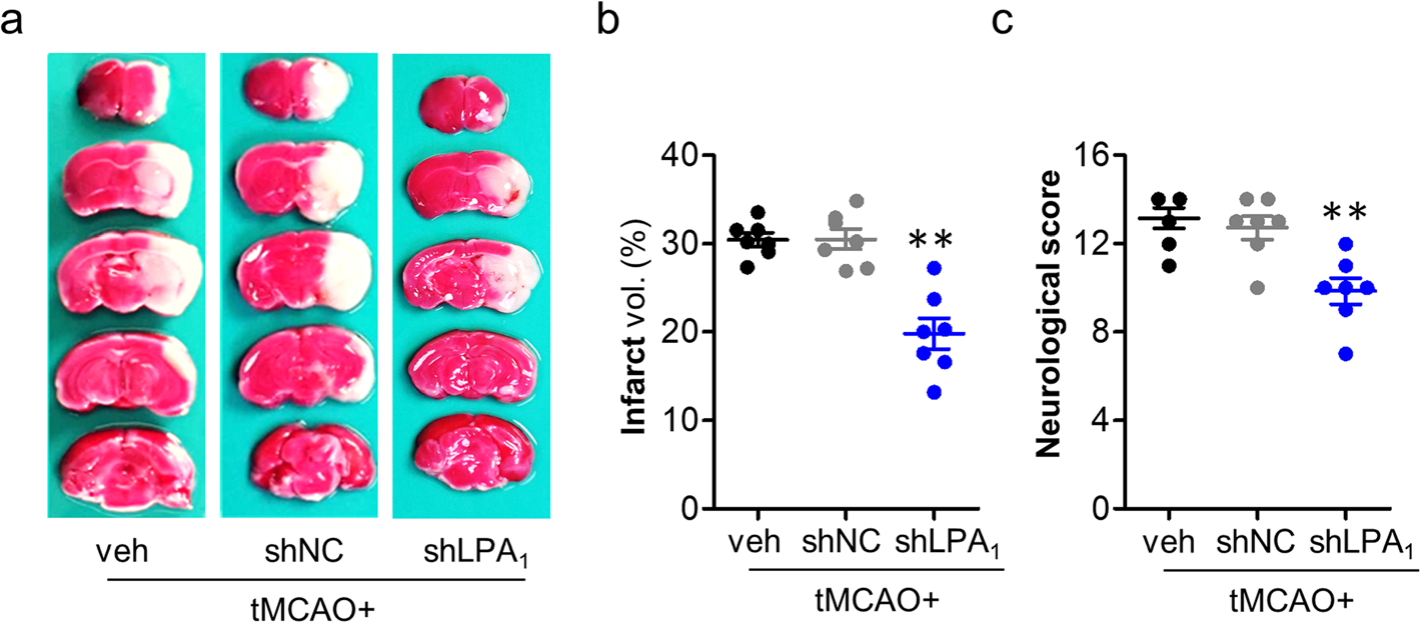
LPA_1_ knockdown reduces brain damage in tMCAO-challenged mice. LPA_1_ shRNA (shLPA_1_) and non-target control shRNA (shNC) particles were injected into the ventricle. One week later, mice were challenged with tMCAO. Brain damage was assessed at 1 day after tMCAO challenge. **a**–**c** Effects of LPA_1_ knockdown on infarct volume (**a**, **b**) and neurological function (**c**) were determined. Representative images of TTC-stained brain slices (**a**) and quantification of brain infarction (**b**). Neurological score indicating neurological functions (**c**). *n* = 7 mice per group. **p < 0.01 versus non-target control lentivirus injected tMCAO mice (tMCAO+shNC)

Having established benefits of pharmacological inhibition of LPA_1_ in cerebral ischemia, we next sought to determine whether brain damage in cerebral ischemia could also be reduced upon LPA_1_ knockdown using its specific shRNA lentivirus. Intracerebroventricular injection of LPA_1_ shRNA lentivirus particles caused its knockdown in the brain (Additional file 3: Figure S3). Under this condition, tMCAO-induced brain damage such as brain infarction (Fig. 2a, b) and neurological dysfunction (Fig. 2c) was markedly reduced compared to that in the tMCAO group infected with non-target control lentivirus. These data independently support the possible pathogenic role of LPA_1_ in cerebral ischemia.

**Fig. 3:**
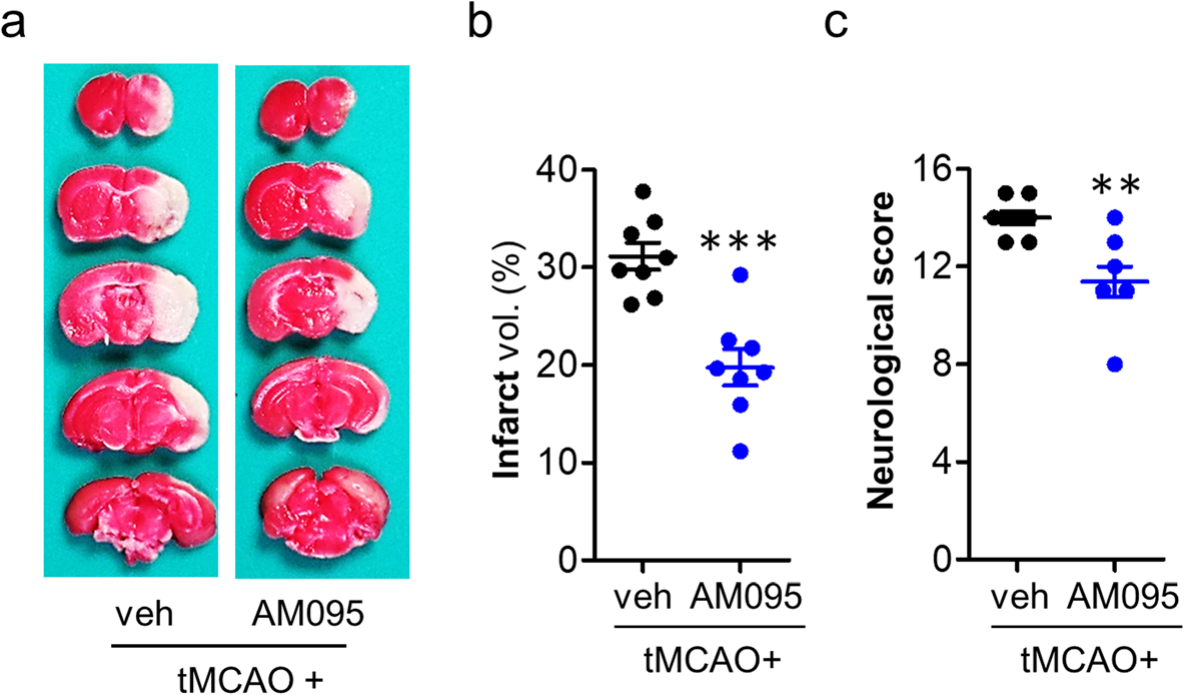
LPA_1_ antagonism reduces brain damage at 3 days after tMCAO challenge. Mice were challenged with tMCAO. AM095 (30 mg/kg, p.o.) was then administered immediately after reperfusion. Brain damage was assessed at 3 days after tMCAO. **a**–**c** Effects of AM095 on infarct volume (**a**, **b**) and neurological function (**c**) were determined. Representative images of TTC-stained brain slices (**a**) and quantification of brain infarction (**b**). Neurological score indicating neurological functions (**c**). *n* = 8 mice per group. **p < 0.01 and ***p < 0.001 versus vehicle-administered tMCAO mice (tMCAO+veh)

We further addressed whether effects of LPA_1_ antagonism could persist up to 3 days after tMCAO challenge. AM095 administration immediately after reperfusion also significantly attenuated brain infarction (Fig. 3a, b; 31.13 ± 1.40% or 19.76 ± 1.85% in vehicle-treated tMCAO group or AM095-treated tMCAO group) and neurological deficit score (Fig. 3c) at 3 days after tMCAO challenge.

The current in vivo findings suggest that LPA_1_ could be a pathogenic factor in cerebral ischemia, further indicating that LPA_1_ expression might be altered in an ischemic brain. Therefore, we determined the mRNA expression levels of LPA_1_ in the ischemic brain at 1 day after tMCAO challenge. Unexpectedly, tMCAO challenge reduced the mRNA expression levels of LPA_1_ (Additional file 4: Figure S4).

### LPA_1_ regulates microglial activation and proliferation in the ischemic brain after tMCAO

**Fig. 4:**
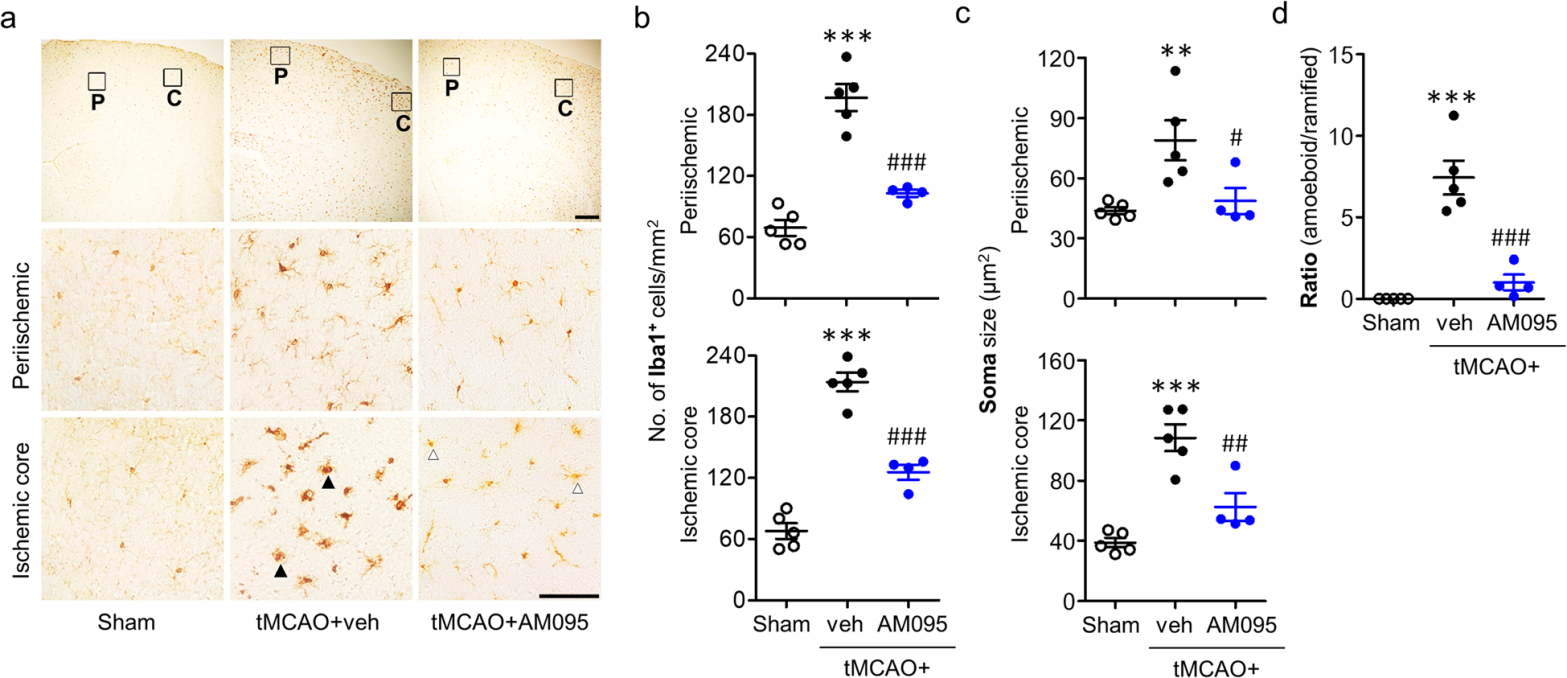
LPA_1_ antagonism reduces microglial activation in the ischemic brain at 1 day after tMCAO challenge. Mice were challenged with tMCAO, and AM095 (30 mg/kg, p.o.) was administered immediately after reperfusion. Microglial activation was assessed at 1 day after tMCAO challenge by Iba1 immunohistochemistry. **a** Representative images of Iba1-immunopositive cells in the periischemic (P) and the ischemic core (C) regions. Diagram boxes display the cerebral area where images in middle and bottom panels are acquired. Scale bars, 200 μm (top panels) and 50 μm (middle and bottom panels). Open arrowheads indicate ramified microglia, and closed arrowheads indicate amoeboid microglia. **b** Quantification of the number of Iba1-immunopositive cells in both regions. **c** Quantification of soma size of Iba1-immunopositive cells in both regions. **d** Quantification of the number of morphologically transferred microglial cells in the ischemic core region (ramified microglia to amoeboid microglia transformation). *n* = 5 (sham), 5 (tMCAO+veh), and 4 (tMCAO+AM095). **p < 0.01 and ***p < 0.001 versus sham. ^#^*p* < 0.05, ^##^*p* < 0.01, and ^###^*p* < 0.001 versus vehicle-administered tMCAO mice (tMCAO+veh)

**Fig. 5:**
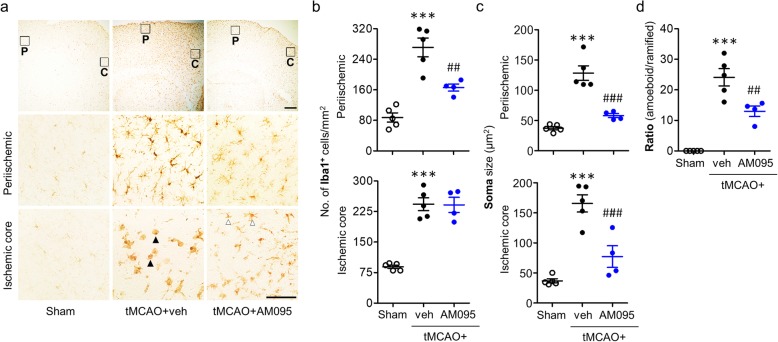
LPA_1_ antagonism reduces microglial activation in the ischemic brain at 3 days after tMCAO challenge. Mice were challenged with tMCAO, and AM095 (30 mg/kg, p.o.) was administered immediately after reperfusion. Microglial activation was assessed at 3 days after tMCAO challenge by Iba1 immunohistochemistry. **a** Representative images of Iba1-immunopositive cells in the periischemic (P) and the ischemic core (C) regions. Diagram boxes display the cerebral area where images in middle and bottom panels are acquired. Scale bars, 200 μm (top panels) and 50 μm (middle and bottom panels). Open arrowheads indicate ramified microglia, and closed arrowheads indicate amoeboid microglia. **b** Quantification of the number of Iba1-immunopositive cells in both regions. **c** Quantification of soma size of Iba1-immunopositive cells in both regions. **d** Quantification of the number of morphologically transferred microglial cells in the ischemic core region (ramified microglia to amoeboid microglia transformation). *n* = 5 (sham), 5 (tMCAO+veh), and 4 (tMCAO+AM095). ***p < 0.001 versus sham. ^##^*p* < 0.01 and ^###^*p* < 0.001 versus vehicle-administered tMCAO mice (tMCAO+veh)

LPA_1_ has been demonstrated to be functionally important for microglial activation [6, 26, 27]. The time- and region-dependent microglial activation is a well-characterized pathogenic feature in an ischemic brain [20, 28, 29]. To address whether LPA_1_-mediated brain damage might be associated with microglial activation in cerebral ischemia, we determined Iba1 immunoreactivity, soma size of Iba1-immunopositive cells, and morphological changes of Iba1-immunopositive cells in ischemic brain. Specifically, we analyzed time- and ischemic region-dependent activation of microglia. In vehicle-administered ischemic brain at 1 day after tMCAO, numbers of Iba1-immunopositive cells in both periischemic and ischemic core regions were significantly increased compared to those in the sham group. When mice were treated with AM095 immediately after reperfusion, the observed increase in the number of activated microglia in both regions was markedly reduced (Fig. 4a, b). Similarly, soma size of Iba1-immunopositive cells was increased in both regions of the ischemic brain, which was significantly attenuated by AM095 administration (Fig. 4a, c). In addition, most of Iba1-immunopostive cells in the ischemic core region were amoeboid microglia whereas they were converted into ramified upon AM095 administration (Fig. 4a, d). We also determined whether the reduced microglial activation through the suppression of LPA_1_ activity in the ischemic brain persisted up to 3 days after tMCAO. AM095 administration reduced the number of Iba1-immunopositive cells in periischemic region along with the thinner cell body of microglia (Fig. 5a–c). Interestingly, in the ischemic core region, LPA_1_ was closely associated with morphological change of activated microglia. There was no significant difference in the number of Iba1-immunopositive cells in the ischemic core region between AM095-treated and vehicle-treated groups (Fig. 5a, b). However, there was a clear reduction in both the soma size of Iba1-immunopositive cells (Fig. 5a, c) and the number of amoeboid microglia (Fig. 5a, d) by AM095 administration in this region. These data were recapitulated upon LPA_1_ knockdown. LPA_1_ knockdown significantly reduced the number of Iba1-immunopositive cells and their soma size in both regions at 1 day after tMCAO challenge (Additional file 5: Figure S5a–c). In the ischemic core region, LPA_1_ knockdown markedly reduced the number of amoeboid microglia (Additional file 5: Figure S5a, d). At 3 days after tMCAO challenge, LPA_1_ knockdown reduced the number of Iba1-immunopositive cells in the periischemic region, but not in the ischemic core region (Additional file 6: Figure S6a, b), as AM095 treatment did. However, LPA_1_ knockdown reduced the soma size of Iba1-immunopositive cells in both periischemic and ischemic core regions (Additional file 6: Figure S6a, c). Additionally, in the ischemic core region, LPA_1_ knockdown markedly reduced the number of amoeboid microglia (Additional file 6: Figure S6a, b, d). Collectively, these data indicate that microglial activation could be a LPA_1_-relevant pathogenic event in cerebral ischemia.

**Fig. 6:**
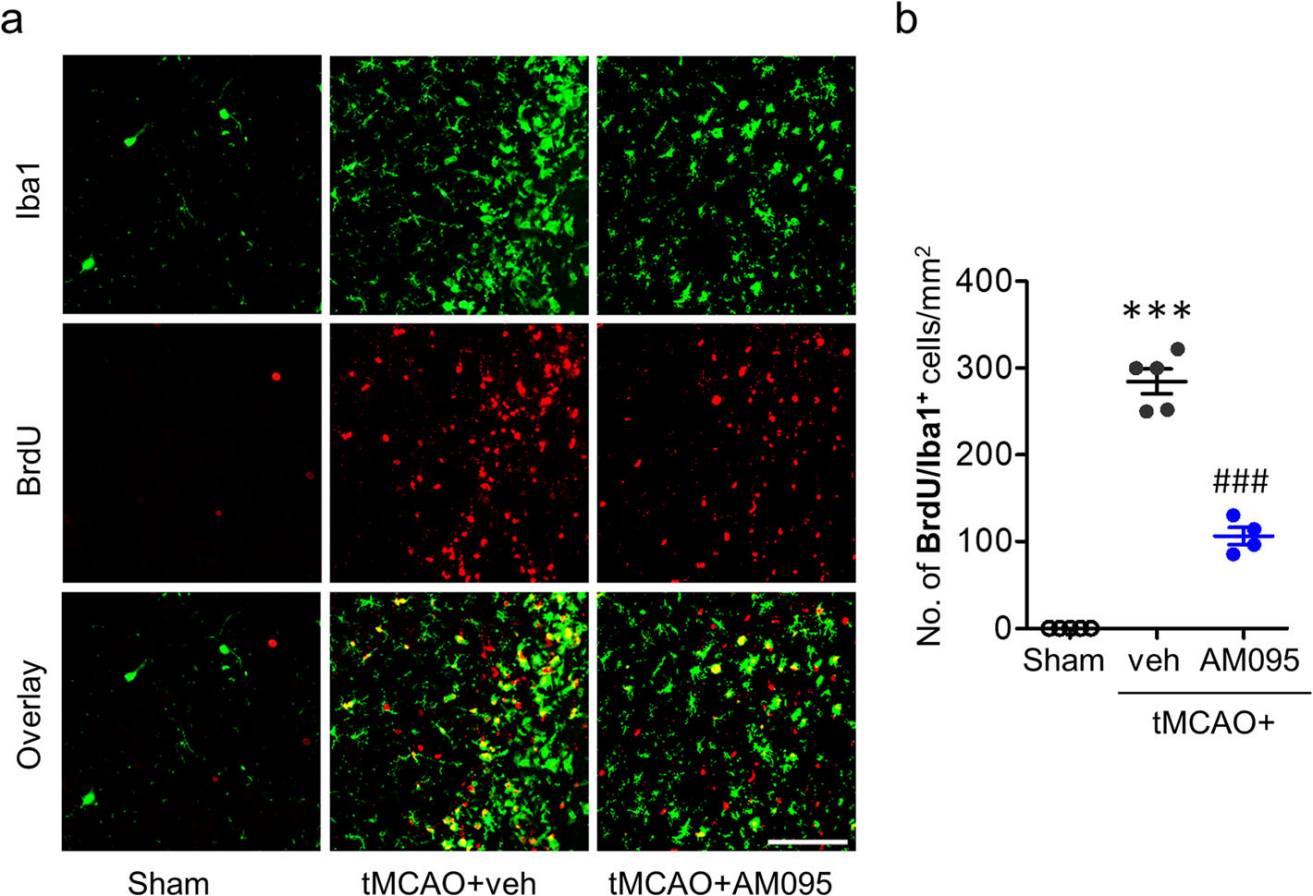
LPA_1_ antagonism reduces microglial proliferation in the ischemic brain at 3 days after tMCAO challenge. Mice were challenged with tMCAO, and AM095 (30 mg/kg, p.o.) was administered immediately after reperfusion. BrdU (50 mg/kg in PBS) was administered twice daily at 12 h interval on days 2 and 3 after tMCAO challenge. Microglial proliferation was assessed at 3 days after tMCAO challenge by double immunofluorescence labeling against BrdU and Iba1. **a** Representative images of Iba1/BrdU-double immunopositive cells in the marginal zone (area between the periischemic and the ischemic core regions) of the ischemic brain. Scale bar, 50 μm. **b** Quantification of the number of Iba1/BrdU-double immunopositive cells. *n* = 5 (sham), 5 (tMCAO+veh), and 4 (tMCAO+AM095). ***p < 0.001 versus sham. ^###^*p* < 0.001 versus vehicle-administered tMCAO mice (tMCAO+veh)

To address whether LPA_1_ signaling was involved in microglial proliferation occurring in an ischemic brain, we determined BrdU signals in activated microglia through double immunofluorescence labeling for the injured area between periischemic and ischemic core regions at 3 days after tMCAO challenge. The number of Iba1/BrdU-double positive cells in AM095-administered mice was dramatically decreased as compared to that in vehicle-treated mice (Fig. 6), which was reaffirmed upon LPA_1_ knockdown (Additional file 7: Figure S7). These data demonstrate that LPA_1_ also regulates microglial proliferation in the ischemic brain.

Besides microglia, activated astrocytes also play key roles to modulate inflammatory responses in an ischemic brain [30]. To determine whether LPA_1_-mediated brain damage might be associated with astrogliosis, we analyzed GFAP immunoreactivity in the ischemic brain at both 1 day and 3 days after tMCAO challenge. In vehicle-administered group, the number of GFAP-immunopositive cells was increased mainly in the penumbra area of the corpus callosum at both time points (Additional file 8: Figure S8a–c). LPA_1_ antagonism significantly attenuated the observed astrogliosis (Additional file 8: Figure S8a–c). In addition, to determine whether LPA_1_ could regulate the proliferation of activated astrocytes in an ischemic brain, we assessed BrdU signals in activated astrocytes through double immunofluorescence labeling for the injured area in the ischemic penumbra region at 3 days after tMCAO challenge. Although fewer GFAP/BrdU-double positive cells than Iba1/BrdU-double positive cells were present in the ischemic penumbra region, the number of GFAP/BrdU-double positive cells was significantly increased compared to that in the sham group (Additional file 8: Figure S8d, e). The number of GFAP/BrdU-double positive cells in AM095-administered mice was significantly decreased as compared to that in vehicle-treated mice (Additional file 8: Figure S8d, e), demonstrating that LPA_1_ also regulated the proliferation of activated astrocytes in the ischemic brain.

### LPA_1_ regulates proinflammatory responses in the ischemic brain after tMCAO

**Fig. 7:**
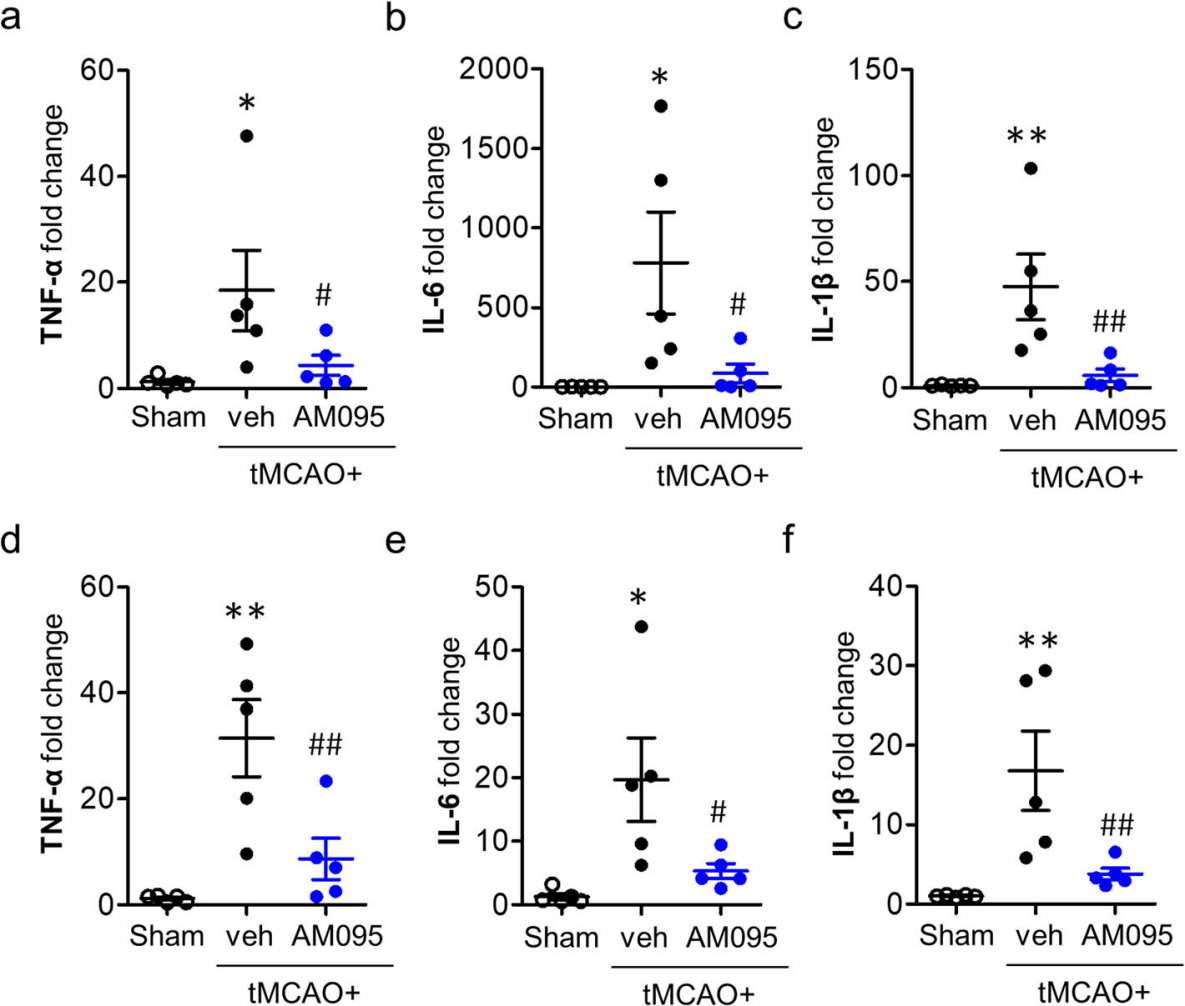
LPA_1_ antagonism attenuates expression levels of proinflammatory cytokines in the ischemic brain after tMCAO challenge. Mice were challenged with tMCAO. AM095 (30 mg/kg, p.o.) was administered immediately after reperfusion. Total RNA was extracted from the ipsilateral brain hemisphere at 1 and 3 days after tMCAO challenge, and mRNA expression levels of proinflammatory cytokines (TNF-α, IL-6, and IL-1β) were determined by qRT-PCR analysis. Changes in expression levels of proinflammatory cytokines at day 1 (**a**–**c**) and at day 3 (**d**–**f**) are shown. *n* = 5 mice per group. *p < 0.05 and **p < 0.01 versus sham. ^#^*p* < 0.05 and ^##^*p* < 0.01 versus vehicle-administered tMCAO mice (tMCAO+veh)

Proinflammatory responses of immune cells including microglia occur in the brain following ischemic injury [31, 32]. To identify whether LPA_1_ might regulate proinflammatory responses in an ischemic brain, expression levels of relevant cytokines were determined by qRT-PCR analysis. Suppressing LPA_1_ activity with AM095 in the ischemic brain significantly attenuated mRNA expression levels of TNF-α, IL-6, and IL-1β at 1 day after tMCAO challenge (Fig. 7a–c). These results were also reproduced at 3 days after tMCAO challenge (Fig. 7d–f). To further identify whether LPA_1_ might regulate anti-inflammatory responses in an ischemic brain, expression levels of anti-inflammatory cytokines (IL-4, TGF-β1, and IL-10) were determined at both 1 day and 3 days following tMCAO challenge (Additional file 9: Figure S9). Expression levels of these cytokines were not altered at 1 day after tMCAO challenge, and AM095 administration did not affect expression levels of them (Additional file 9: Figure S9a–c). But, at 3 days after tMCAO challenge, AM095 administration significantly increased the mRNA expression levels of IL-4, whereas it decreased the mRNA expression levels of TGF-β1 with no significant changes in IL-10 expression (Additional file 9: Figure S9d–f). These data strongly indicate that LPA_1_ could mainly promote proinflammatory responses in the brain after ischemic injury.

**Fig. 8:**
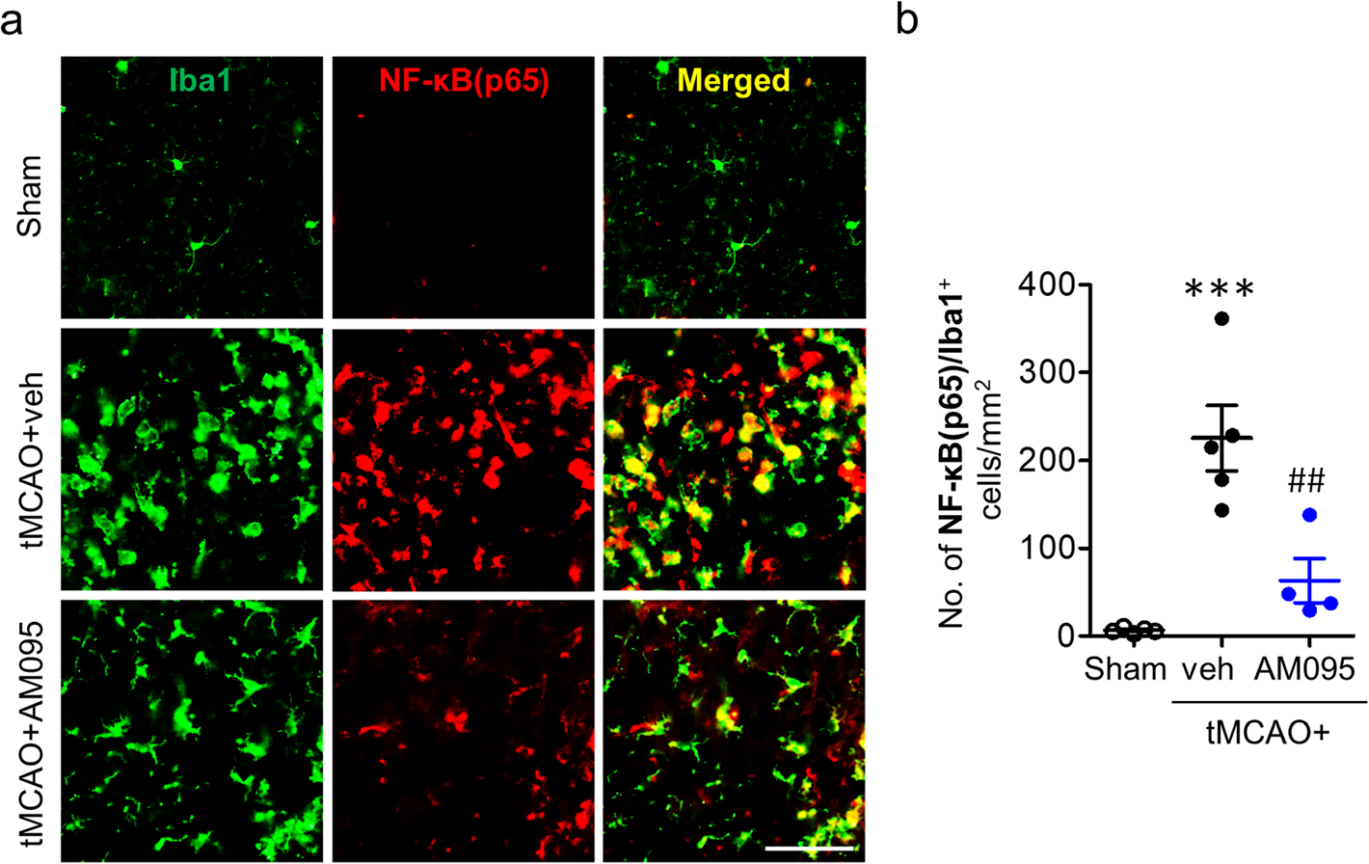
LPA_1_ antagonism reduces NF-κB expression in activated microglia in post-ischemic brain at 3 days after tMCAO challenge. Mice were challenged with tMCAO. AM095 (30 mg/kg, p.o.) was administered immediately after reperfusion. The expression of NF-κB in activated microglia was assessed at 3 days after tMCAO challenge by double immunofluorescence labeling against NF-κB(p65) and Iba1. **a** Representative images of NF-κB(p65)/Iba1-immunopositive cells in ischemic core regions. Scale bar, 50 μm. **b** Quantification of the number of NF-κB(p65)/Iba1-double immunopositive cells. *n* = 5 (sham), 5 (tMCAO+veh), and 4 (tMCAO+AM095). ***p < 0.001 versus sham. ^##^*p* < 0.01 versus vehicle-administered tMCAO mice (tMCAO+veh)

**Fig. 9:**
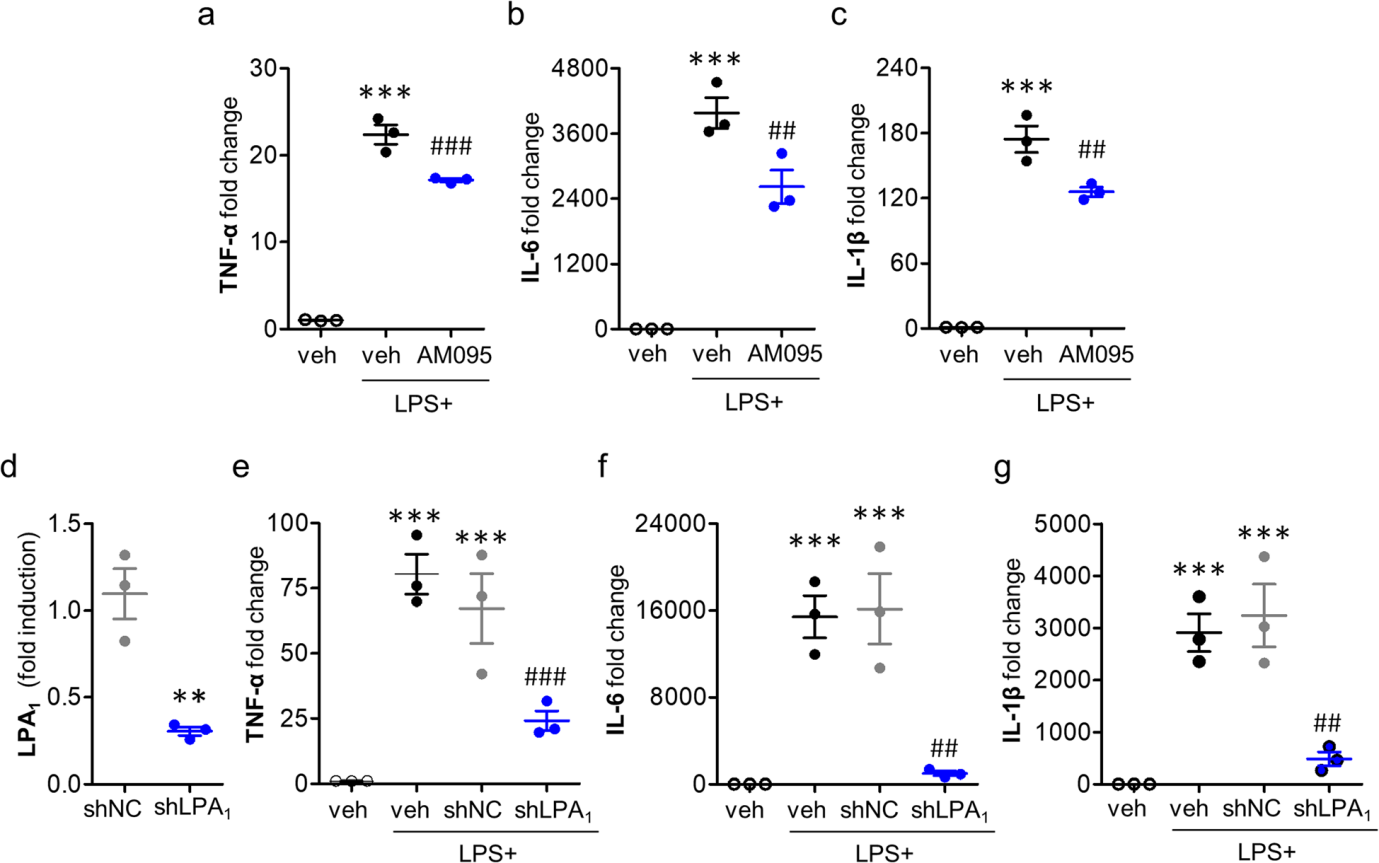
Suppressing LPA_1_ activity attenuates LPS-upregulated mRNA expression levels of proinflammatory cytokines in mouse primary microglia. Primary microglia were stimulated with LPS (100 ng/ml). Thirty minutes prior to LPS exposure, cells were treated with AM095 (2 μM in 0.1% DMSO). Alternatively, primary microglia were stimulated with LPS at 2 days after transfection with lentivirus particles bearing LPA_1_ shRNA (shLPA_1_) or non-target control shRNA (shNC). Total RNA was extracted at 24 h after LPS exposure, and mRNA expression levels of proinflammatory cytokines were determined by qRT-PCR analysis. **a**–**c** Changes in mRNA expression levels of TNF-α, IL-6, and IL-1β. *n* = 3 per group. ***p < 0.001 versus the vehicle-treated cells. ^##^*p* < 0.01 and ^###^*p* < 0.001 versus LPS-stimulated cells. **d**–**g** Effects of LPA_1_ knockdown (**d**) on the mRNA expression levels of TNF-α, IL-6, and IL-1β (**e**–**g**). *n* = 3 per group. **p < 0.01 versus cells infected with non-target control shRNA lentivirus (shNC) in **d**. ***p < 0.001 versus vehicle-treated cells in **e**–**g**. ^##^*p* < 0.01 and ^###^*p* < 0.001 versus LPS-stimulated cells infected with non-target control shRNA lentivirus (shNC+LPS) in **e**–**g**

In addition to evidence on gene expression, the role of LPA_1_ in proinflammatory responses after ischemic challenge was reaffirmed by determining NF-κB activation because proinflammatory responses were closely linked to activation of NF-κB signaling [33, 34]. Suppressing LPA_1_ activity with AM095 administration significantly reduced the number of NF-κB (p65)-immunopositive cells (Fig. 8). These results reaffirmed that LPA_1_ was involved in promoting proinflammatory responses in the ischemic brain. More importantly, most of NF-κB (p65) signals in the ischemic brain were observed in Iba1-immunopositive cells (Fig. 8), demonstrating that NF-κB activation occurred in activated microglia. These results further implicate that activated microglia could be the main loci for LPA_1_-dependent proinflammatory responses in the ischemic brain. This notion from in vivo finding was further affirmed using LPS-stimulated primary microglia in vitro because LPS has been widely used to induce proinflammatory responses of microglia [27, 35]. When AM095-treated primary microglia were exposed to LPS, mRNA expression levels of TNF-α, IL-6, and IL-1β were significantly decreased (Fig. 9a–c). In consistent with AM095 treatment, LPA_1_ knockdown in primary microglia (Fig. 9d) markedly decreased the expression levels of TNF-α, IL-6, and IL-1β following LPS stimulation (Fig. 9e–g).

### LPA_1_ influences MAPK and PI3K/Akt activation in the ischemic brain

**Fig. 10:**
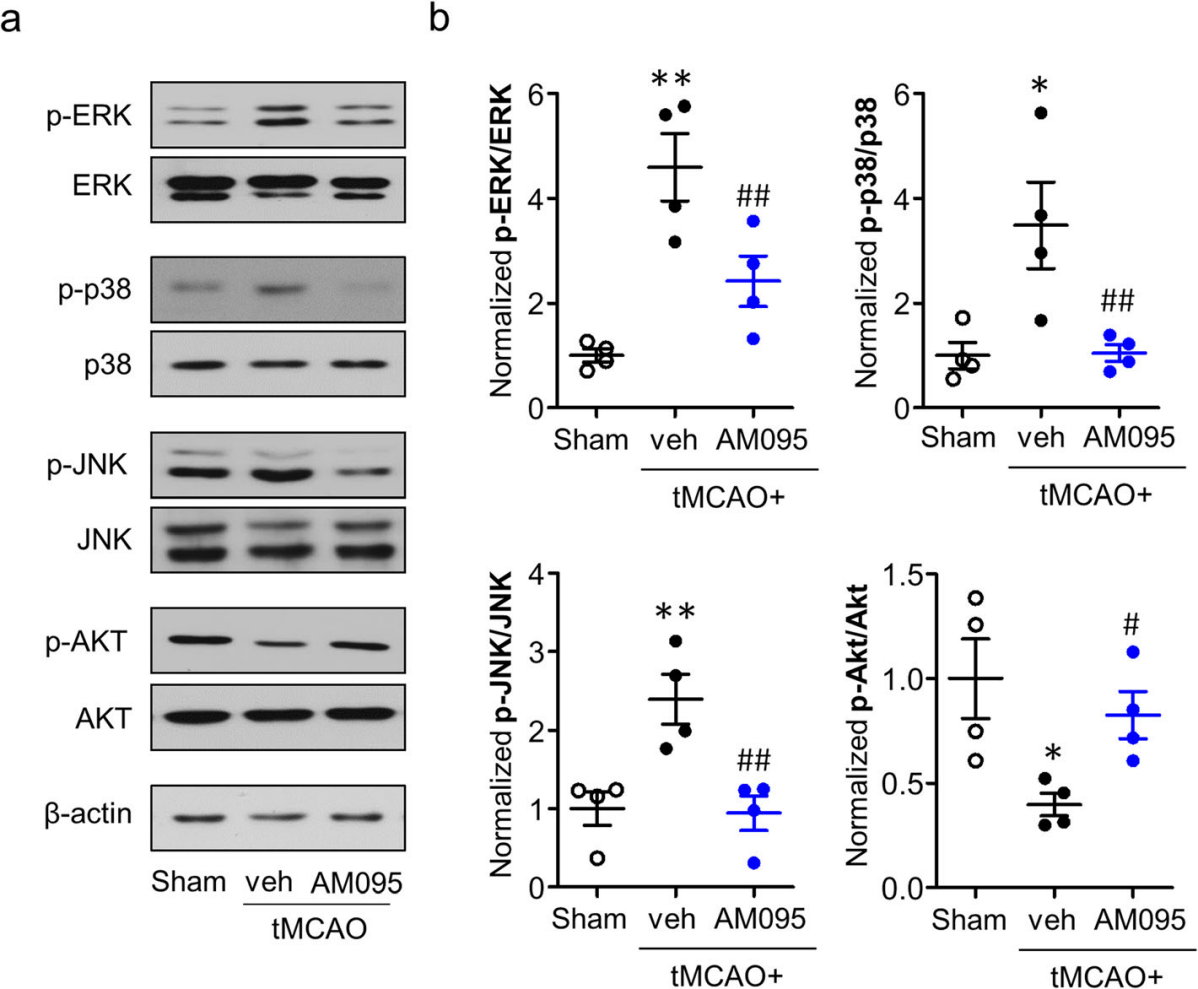
LPA_1_ antagonism attenuates MAPK phosphorylation and enhances Akt phosphorylation in the ischemic brain after tMCAO challenge. Mice were challenged with tMCAO, and AM095 (30 mg/kg, p.o.) was administered immediately after reperfusion. Total protein was extracted from the ipsilateral brain hemisphere at 1 day after tMCAO challenge. Phosphorylation levels of MAPKs and Akt were then determined by Western blot analysis. Representative Western blots (**a**) and their quantification (**b**). *n* = 4 mice per group. *p < 0.05 and **p < 0.01 versus sham. ^#^*p* < 0.05 and ^##^*p* < 0.01 versus vehicle-administered tMCAO mice (tMCAO+veh)

In neuroinflammatory circumstances, LPA_1_ activates several effector pathways including MAPKs and PI3K/Akt [15]. These signaling pathways are also linked to proinflammatory responses in the CNS [36, 37]. To verify whether LPA_1_ also influenced these effector pathways in the ischemic brain, we determined the activation of MAPKs and PI3K/Akt signaling pathways at 1 day after tMCAO challenge by Western blot analysis. Expression levels of phosphorylated ERK1/2, p38, and JNK MAPKs were significantly increased in the ischemic hemispheres of the vehicle-administered mice (Fig. 10). These increases were significantly attenuated by AM095 administration (Fig. 10). In case of PI3K/Akt pathways, Akt phosphorylation was significantly reduced in the brains of vehicle-administered mice whereas AM095 administration reversed this suppression (Fig. 10). These data demonstrated that LPA_1_ modulated MAPKs and PI3K/Akt as downstream pathways to mediate brain damage in cerebral ischemia.

## Discussion

LPA_1_ is of great interest because therapeutic advances targeting it have been made through ongoing clinical trials to treat several diseases, including idiopathic pulmonary fibrosis (ClinicalTrials.gov ID: NCT01766817) and psoriasis (ClinicalTrials.gov ID: NCT02763969) [4]. According to these advances, validating a therapeutic potential to target LPA_1_ in diverse disease types has attracted more interest [4]. In this study, we validated the potential in transient focal cerebral ischemia by providing direct evidence that LPA_1_ was a critical factor for brain damages in an ischemic brain. Distinctly, suppressing LPA_1_ activity resulted in the reduction of brain infarction and improvement of neurological function. As a key pathogenic event, we proved that LPA_1_ regulated microglial activation towards accelerating immune responses in the brain. Finally, MAPK and PI3K/Akt were identified as effector pathways for the role of LPA_1_ in focal cerebral ischemia.

The best-studied LPA receptor subtype is LPA_1_. Its pathogenic roles in the CNS are linked into spinal cord injury [6] and neuropathic pain [17, 38]. In cerebral ischemia, the exact role of receptor-mediated LPA signaling has not been reported yet. However, a few studies have indicated that receptor-mediated LPA signaling has a certain role in ischemic conditions [17, 39, 40]. One study has reported that the amount of the ligand, LPA, is increased in plasma from ischemic patients with non-valvular atrial fibrillation [39]. Another study has revealed that LPA_1_ knockdown can reduce hypoxia-induced retinal cell death [40]. Additionally, in rodent models, LPA_1_ deletion reduced neuropathic pain responses in mice challenged by cerebral ischemia [17, 18], but it is unclear whether LPA_1_ contributes to ischemic brain damage directly, such as brain infarction and neurological functional deficit. In the present study, we reported that LPA_1_ was critical for direct brain damages following transient focal cerebral ischemia for the first time. We found that pharmacological or genetic suppression of LPA_1_ activity significantly attenuated tMCAO-induced brain infarction and neurological deficits. Despite this clear role of LPA_1_ in ischemic brain damage, its expression at mRNA levels was, unexpectedly, downregulated in the ischemic brain. Similar unexpected results were observed for S1P_1_ [20, 41]. Either pharmacological or genetic suppression of S1P_1_ activity significantly attenuated tMCAO-induced brain infarction and neurological deficits [20]. But, mRNA expression levels of S1P_1_ were downregulated in the ischemic brain [41], likely LPA_1_ expression (the current study).

Excessive activation of microglia has been regarded as a core pathogenesis of a variety of brain diseases [42, 43]. In cerebral ischemia, this activation can lead to brain damages [16]. We found that roles of LPA_1_ were possibly linked into microglial activation and proliferation. In fact, it has been well known that LPA signaling regulates various biological functions of microglia, including migration, metabolism, membrane dynamics, ion conductance, and growth factor production [3]. Additionally, intraspinal injection of LPA can trigger microglial activation as demonstrated by the upregulation of activated microglial markers (i.e., Iba1) [6, 44] and morphological transformation from ramified microglia to amoeboid microglia. Importantly, at least two LPA receptor subtypes have been identified to be able to regulate microglial activation: LPA_1_ [6, 15] and LPA_5_ [45, 46]. In particular, LPA_1_ regulates microglial activation in injured spinal cord [6] and septic brain [15]. Suppressing LPA_1_ activity can improve cell survival in oligodendrocytes [6] and attenuate microglial activation and their morphological transformation into toxic forms, amoeboid microglia, in LPS-induced septic brain [15]. Both independent studies strongly indicate that LPA_1_ exerts its pathological functions through microglial activation. In line with this notion, in the current study, suppressing LPA_1_ activity reduced microglial activation (their numbers and soma size), their proliferation, and morphological transformation of microglia from ramified microglia to amoeboid microglia in the ischemic brain.

Detrimental roles of activated microglia are closely linked to their proinflammatory responses following ischemic challenge. Activated microglia that can potentiate secondary brain damage could be the desired target for therapeutic intervention in the treatment of neuroinflammatory disorders such as cerebral ischemia [16, 32]. In the present study, suppressing LPA_1_ activity attenuated proinflammatory responses in the ischemic brain, particularly in activated microglia. The latter was supported by in vivo and in vitro findings, in which suppressing LPA_1_ activity attenuated the activation of microglial NF-κB, a critical regulator of proinflammatory responses, in the ischemic brain and downregulated the expression levels of proinflammatory cytokines in primary microglia stimulated with LPS, a well-known inducer of proinflammatory responses [27, 35]. Among LPA receptor subtypes, LPA_5_ has been identified to be able to drive microglia towards their proinflammatory phenotype in a previous study utilizing LPS-primed BV2 murine microglia [46]. Besides LPA_5_ [46], our current study identified LPA_1_ as another LPA receptor subtype that influenced proinflammatory responses of microglia. This was also supported by our previous independent study in which LPA_1_ promoted TNF-α production in septic brain and LPS-stimulated rat primary microglia [15]. Taken together, these data demonstrate that LPA_1_ not only controls microglial activation and proliferation, but also controls proinflammatory responses of microglia, indicating that role of LPA_1_ in ischemic brain damage could be mediated through promoting the proinflammatory responses of activated microglia, at least in part.

In addition to microglial activation, astrogliosis is another core pathogenesis of cerebral ischemia [30]. Although the role of LPA_1_ in cerebral ischemia-induced astrogliosis was not reported previously, it could be the regulator of astrogliosis in the ischemic brain as well. In fact, a previous study indicated that LPA_1_ could modulate astrogliosis in the injured spinal cord of mouse with sciatic nerve ligation-induced neuropathic pain [47]. This previous study demonstrated that Ki16425, (an LPA_1/3_ antagonist) reduced astrogliosis in the injured spinal cord [47]. Given data for the absence of LPA_3_ in astrocytes, this previous report further indicates that LPA_1_ could modulate astrogliosis in the injured spinal cord [47]. Therefore, LPA_1_ could also influence astrogliosis in an ischemic brain. Indeed, LPA_1_ antagonism significantly attenuated astrogliosis and astrocytic proliferation in the ischemic brain. The latter was further supported by another previous study, in which LPA-triggered proliferation of cultured astrocytes was completely abolished upon LPA_1_ deletion [48].

It is well-known that LPA_1_ signaling can mediate different downstream effector pathways, such as MAPKs and PI3K/Akt, through G_αi/o_, G_αq/11_, and G_α12/13_ that mediate multiple biological functions [3]. Furthermore, LPA_1_ influenced ERK1/2 effector pathway in both LPS-induced septic brain and LPS-stimulated cultured microglia [15]. In both conditions, Akt activation was not altered [15]. In cultured cells, p38 was associated with microglial activation without any relevance to LPA_1_ [15]. Unlike the previous data, the current in vivo findings showed that the role of LPA_1_ in cerebral ischemia was linked to MAPK and PI3K/Akt because suppressing LPA_1_ activity in the ischemic brain decreased the phosphorylation of ERK1/2, p38, and JNK whereas it increased Akt phosphorylation. The difference between previous and current findings might be from different experimental systems. Notably, MAPK activation was critical for promoting proinflammatory responses. Persistent activation of ERK1/2, p38, and JNK pathways can activate NF-κB-regulated transcription [49–52]. MAPK-mediated NF-κB activation leads to the secretion of proinflammatory mediators such as cytokines and chemokines [53–55] that are mainly associated with proinflammatory responses. P13K/Akt pathway is also closely related to proinflammatory responses, although Akt activation has dual nature. It not only potentiates anti-inflammatory responses, but also counters proinflammatory responses through a negative regulation of NF-κB activation [33]. Taken together, these results demonstrate that LPA_1_ participates in the brain damage of cerebral ischemia through PI3K/Akt and MAPK effector pathways.

## Conclusions

In conclusion, results of the present study suggest that LPA_1_ could act as a novel pathogenic factor in cerebral ischemia through mediating brain damage. In the ischemic brain, LPA_1_ promotes glial activation (particularly including microglial activation and subsequent proinflammatory responses) and influences MAPK and PI3K/Akt pathways. The current in vivo evidence clearly demonstrates that suppressing LPA_1_ activity is effective for acute brain injury in cerebral ischemia, but it remains unclear whether this effect is linked to a long-term protection. Therefore, it would be tempting to address long-term neuroprotective effects by LPA_1_ antagonism as an independent future study. Moreover, targeting LPA_1_ could be an appealing strategy for the treatment of other microglia-mediated neuroinflammatory disorders, including Parkinson’s disease, Alzheimer’s disease, and multiple sclerosis.

## Additional files

Additional file 1: **Figure S1.** Scheme of experimental procedure. (a) Experimental scheme to determine effects of AM095 administration immediately after reperfusion. (b) Experimental scheme to determine effects of AM095 administration at 1 h prior to tMCAO challenge. (c) Experimental scheme to determine effects of LPA_1_ knockdown with LPA_1_ shRNA lentivirus. (PPTX 38 kb)

Additional file 2: **Figure S2.** AM095 pretreatment reduces brain damage in tMCAO-challenged mice. Mice were challenged with tMCAO. AM095 (30 mg/kg, p.o.) was administered at 1 h prior to tMCAO challenge. Brain damage was assessed at 1 day after tMCAO. (a-c) Effects of AM095 on infarct volume (a, b) and neurological function (c). Representative images of TTC-stained brain slices (a) and quantification of brain infarction (b). Neurological score indicating neurological functions (c). *n* = 7 mice per group. **p < 0.01 and ***p < 0.001 versus vehicle-administered tMCAO mice (tMCAO+veh). (PPTX 1860 kb)

Additional file 3: **Figure S3.** Infection with LPA_1_ shRNA lentivirus causes significant LPA_1_ knockdown in normal mice brains. Mice were infected with lentivirus for LPA_1_ shRNA (shLPA_1_) or non-target control shRNA (shNC) through intracerebroventricular injection of lentivirus particles. Mice brains were then obtained at 7 days later for total RNA extraction. Changes in mRNA expression levels of LPA_1_ were determined through qRT-PCR analysis. *n* = 5 mice per group. *p < 0.05 versus mice infected with non-target control shRNA (shNC). (PPTX 403 kb)

Additional file 4: **Figure S4.** LPA_1_ gene expression is reduced in post-ischemic brain. Mice were challenged with tMCAO. Total RNA was extracted from the ipsilateral brain hemisphere at 1 day after tMCAO challenge, and mRNA expression levels of LPA_1_ were determined using qRT-PCR. Changes in expression levels of LPA_1_ are shown. *n* = 5 mice per group. *p < 0.05 versus sham group. (PPTX 36 kb)

Additional file 5: **Figure S5.** LPA_1_ knockdown reduces microglial activation in the ischemic brain at 1 day after tMCAO challenge. LPA_1_ shRNA (shLPA_1_) and non-target control shRNA (shNC) particles were injected into the ventricle. One week later, mice were challenged with tMCAO. Microglial activation was assessed at 1 day after tMCAO challenge by Iba1 immunohistochemistry. (a) Representative images of Iba1-immunopositive cells in the periischemic (P) and the ischemic core (C) regions. Diagram boxes display the cerebral area where images in middle and bottom panels are acquired. Scale bars, 200 μm (top panels) and 50 μm (middle and bottom panels). Open arrowheads indicate ramified microglia and closed arrowheads indicate amoeboid microglia in the ischemic core region. (b) Quantification of the number of Iba1-immunopositive cells in both regions. (c) Quantification of soma size of Iba1-immunopositive cells in both regions. (d) Quantification of the number of morphologically transferred microglial cells in the ischemic core region (ramified microglia to amoeboid microglia transformation). *n* = 5 mice per group. ***p < 0.001 versus sham. ^#^*p* < 0.05, ^##^*p* < 0.01, and ^###^*p* < 0.001 non-target control lentivirus injected tMCAO mice (tMCAO+shNC). (PPTX 35 kb)

Additional file 6: **Figure S6.** LPA_1_ knockdown reduces microglial activation in the ischemic brain at 3 days after tMCAO challenge. LPA_1_ shRNA (shLPA_1_) and non-target control shRNA (shNC) particles were injected into the ventricle. One week later, mice were challenged with tMCAO. Microglial activation was assessed at 3 days after tMCAO challenge by Iba1 immunohistochemistry. (a) Representative images of Iba1-immunopositive cells in the periischemic (P) and the ischemic core (C) regions. Diagram boxes display the cerebral area where images in middle and bottom panels are acquired. Scale bars, 200 μm (top panels) and 50 μm (middle and bottom panels). Open arrowheads indicate ramified microglia and closed arrowheads indicate amoeboid microglia in the ischemic core region. (b) Quantification of the number of Iba1-immunopositive cells in both regions. (c) Quantification of soma size of Iba1-immunopositive cells in both regions. (d) Quantification of the number of morphologically transferred microglial cells in the ischemic core region (ramified microglia to amoeboid microglia transformation). *n* = 5 mice per group. **p < 0.01 and ***p < 0.001 versus sham. ^#^*p* < 0.05, ^##^*p* < 0.01, and ^###^*p* < 0.001 versus non-target control lentivirus injected tMCAO mice (tMCAO+shNC). (PPTX 7448 kb)

Additional file 7: **Figure S7.** LPA_1_ knockdown reduces microglial proliferation in the ischemic brain at 3 days after tMCAO challenge. LPA_1_ shRNA (shLPA_1_) and non-target control shRNA (shNC) particles were injected into the ventricle. One week later, mice were challenged with tMCAO. BrdU (50 mg/kg in PBS) was administered twice daily at 12 h interval on days 2 and 3 after tMCAO challenge. Microglial proliferation was assessed at 3 days after tMCAO challenge by double immunofluorescence labeling against BrdU and Iba1. (a) Representative images of Iba1/BrdU-double immunopositive cells in the marginal zone (area between the periischemic and the ischemic core regions) of the ischemic brain. Scale bar, 50 μm. (b) Quantification of the number of Iba1/BrdU-double immunopositive cells. *n* = 5 mice per group. ***p < 0.001 versus sham. ^#^*p* < 0.05 versus non-target control lentivirus injected tMCAO mice (tMCAO+shNC). (PPTX 2734 kb)

Additional file 8: **Figure S8.** LPA_1_ antagonism reduces activation and proliferation of astrocytes in the ischemic brain. Mice were challenged with tMCAO. AM095 (30 mg/kg, p.o.) was administered immediately after reperfusion. Activation of astrocytes was assessed at 1 day and 3 days after tMCAO challenge by GFAP immunohistochemistry. (a) Representative images of GFAP-immunopositive cells in the penumbra area of the corpus callosum. Scale bar, 50 μm. (b, c) Quantification of the number of GFAP-immunopositive cells at 1 day (b) and 3 days (c) after tMCAO challenge. (d, e) Proliferation of astrocytes was assessed at 3 days after tMCAO challenge by double immunofluorescence labeling against BrdU and GFAP. (d) Representative images of GFAP/BrdU-double immunopositive cells in the marginal zone (area between the periischemic and the ischemic core regions) of the ischemic brain. Scale bar, 50 μm. (e) Quantification of the number of GFAP/BrdU-double immunopositive cells. *n* = 5 (sham), 5 (tMCAO+veh), and 4 (tMCAO+AM095). ***p < 0.001 versus sham. ^##^*p* < 0.01 and ^###^*p* < 0.001 versus vehicle-administered tMCAO mice (tMCAO+veh). (PPTX 1610 kb)

Additional file 9: **Figure S9.** LPA_1_ antagonism alters expression levels of few anti-inflammatory cytokines in the ischemic brain after tMCAO challenge. Mice were challenged with tMCAO. AM095 (30 mg/kg, p.o.) was administered immediately after reperfusion. Total RNA was extracted from the ipsilateral brain hemisphere at 1 day and 3 days after tMCAO challenge, and mRNA expression levels of anti-inflammatory cytokines were determined by qRT-PCR analysis. Changes in expression levels of anti-inflammatory cytokines at 1 day (a-c) and at 3 days (d-f) are shown. *n* = 5 mice per group. ***p < 0.001 versus sham. ^##^*p* < 0.01 versus vehicle-administered tMCAO mice (tMCAO+veh). (PPTX 54 kb)

Additional file 10: **Table S1.** Primer sets used for qRT-PCR analysis. (PDF 293 kb)

## Data Availability

All data generated or analyzed during this study are included in this published article (and its additional files).

## Abbreviations

ABC: Avidin and biotinylated complex
BrdU: Bromodeoxyuridine
CNS: Central nervous system
DAB: 3, 3′-Diaminobenzidine tetrahydrochloride
ECL: Enhanced chemiluminescence
IL-10: Interleukin 10
IL-1β: Interleukin 1 beta
IL-4: Interleukin 4
IL-6: Interleukin 6
LPA_1_: Lysophosphatidic acid receptor 1
LPS: Lipopolysaccharides
mNSS: Modified neurological severity score
TGF-β1: Transforming growth factor-beta 1
tMCAO: Transient middle cerebral artery occlusion
TNF-α: Tumor necrosis factor-alpha
TTC: 2,3,5-Triphenyltetrazolium chloride
