# Supplementary material for: Lysophosphatidic acid receptor 1 (LPA1) plays critical roles in microglial activation and brain damage after transient focal cerebral ischemia
Source: J Neuroinflammation. 2019 Aug 20;16:170. doi: 10.1186/s12974-019-1555-8 (PMC6701099; doi:10.1186/s12974-019-1555-8)
Supplement: Supplementary file 1 — Figure S1. Scheme of experimental procedure. (a) Experimental scheme to determine effects of AM095 administration immediately after reperfusion. (b) Experimental scheme to determine effects of AM095 administration at 1 h prior to tMCAO challenge. (c) Experimental scheme to determine effects of LPA1 knockdown with LPA1 shRNA lentivirus. (PPTX 38 kb) [file 12974_2019_1555_MOESM1_ESM.pptx]

## Slide 1
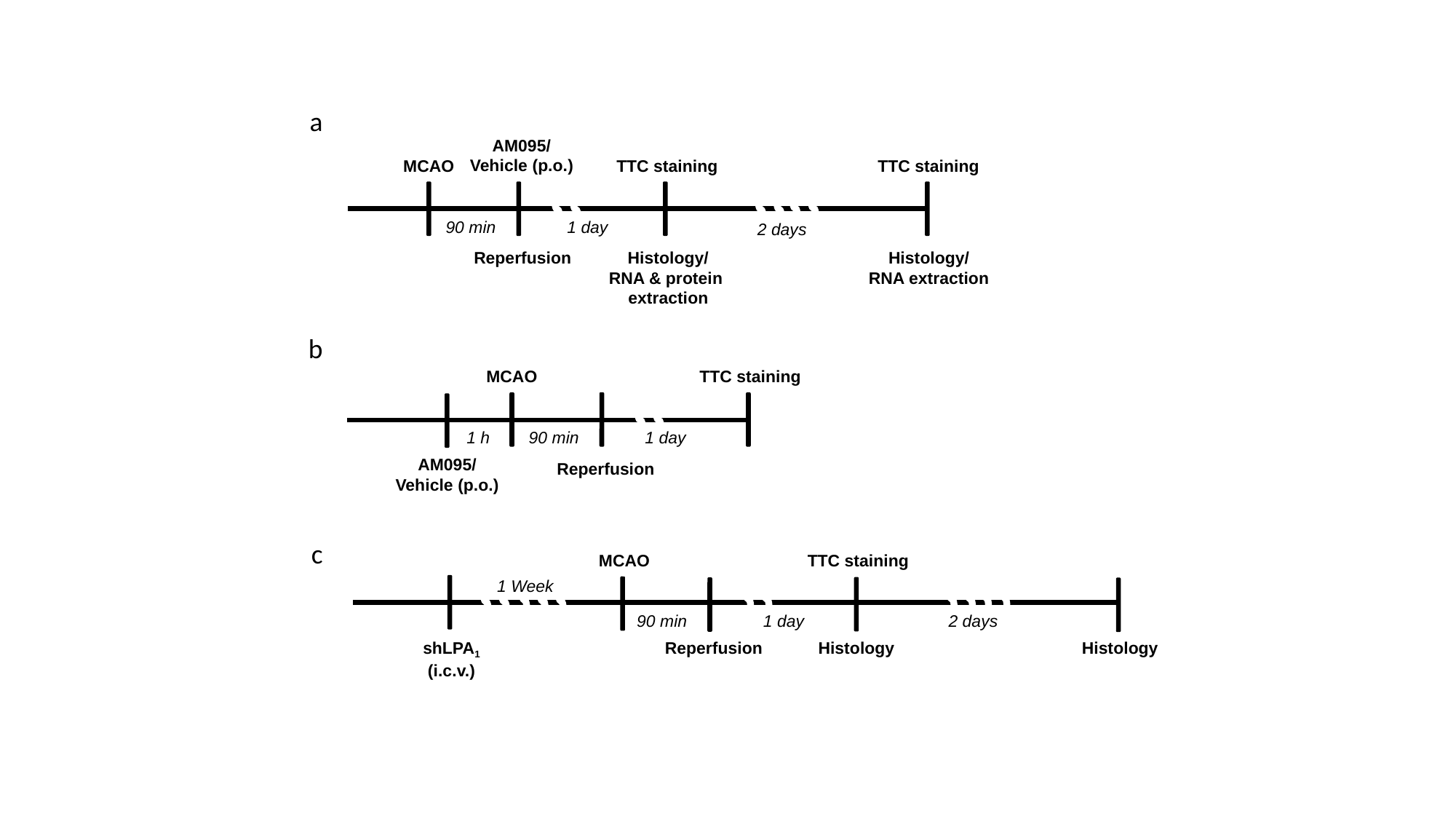

a
AM095/
Vehicle (p.o.)
MCAO
TTC staining
TTC staining
90 min
1 day
2 days
Reperfusion
Histology/
RNA & protein
extraction
Histology/
RNA extraction
b
MCAO
TTC staining
1 h
90 min
1 day
AM095/
Vehicle (p.o.)
Reperfusion
c
MCAO
TTC staining
1 Week
90 min
1 day
2 days
shLPA1
(i.c.v.)
Reperfusion
Histology
Histology
