# Supplementary material for: Lysophosphatidic acid receptor 1 (LPA1) plays critical roles in microglial activation and brain damage after transient focal cerebral ischemia
Source: J Neuroinflammation. 2019 Aug 20;16:170. doi: 10.1186/s12974-019-1555-8 (PMC6701099; doi:10.1186/s12974-019-1555-8)
Supplement: Supplementary file 2 — Figure S2. AM095 pretreatment reduces brain damage in tMCAO-challenged mice. Mice were challenged with tMCAO. AM095 (30 mg/kg, p.o.) was administered at 1 h prior to tMCAO challenge. Brain damage was assessed at 1 day after tMCAO. (a-c) Effects of AM095 on infarct volume (a, b) and neurological function (c). Representative images of TTC-stained brain slices (a) and quantification of brain infarction (b). Neurological score indicating neurological functions (c). n = 7 mice per group. **p < 0.01 and ***p < 0.001 versus vehicle-administered tMCAO mice (tMCAO+veh). (PPTX 1860 kb) [file 12974_2019_1555_MOESM2_ESM.pptx]

## Slide 1
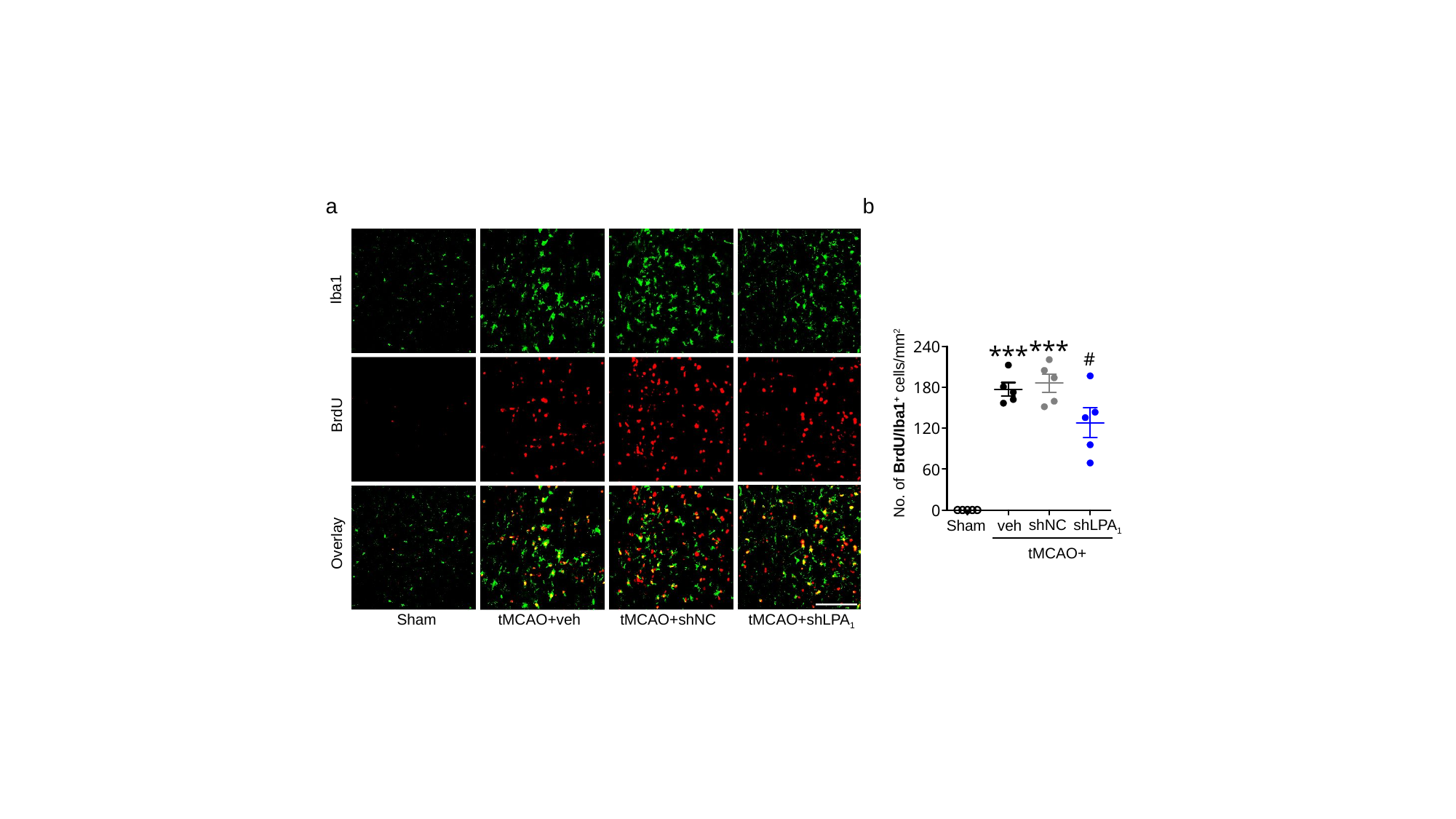

a
b
Iba1
***
***
#
BrdU
No. of BrdU/Iba1+ cells/mm2
shNC
shLPA1
Sham
veh
Overlay
tMCAO+
Sham
tMCAO+veh
tMCAO+shNC
tMCAO+shLPA1
