# Supplementary figures and images for: Lysophosphatidic acid receptor 1 (LPA1) plays critical roles in microglial activation and brain damage after transient focal cerebral ischemia
Source: J Neuroinflammation. 2019 Aug 20;16:170. doi: 10.1186/s12974-019-1555-8 (PMC6701099; doi:10.1186/s12974-019-1555-8)

## Slide 1
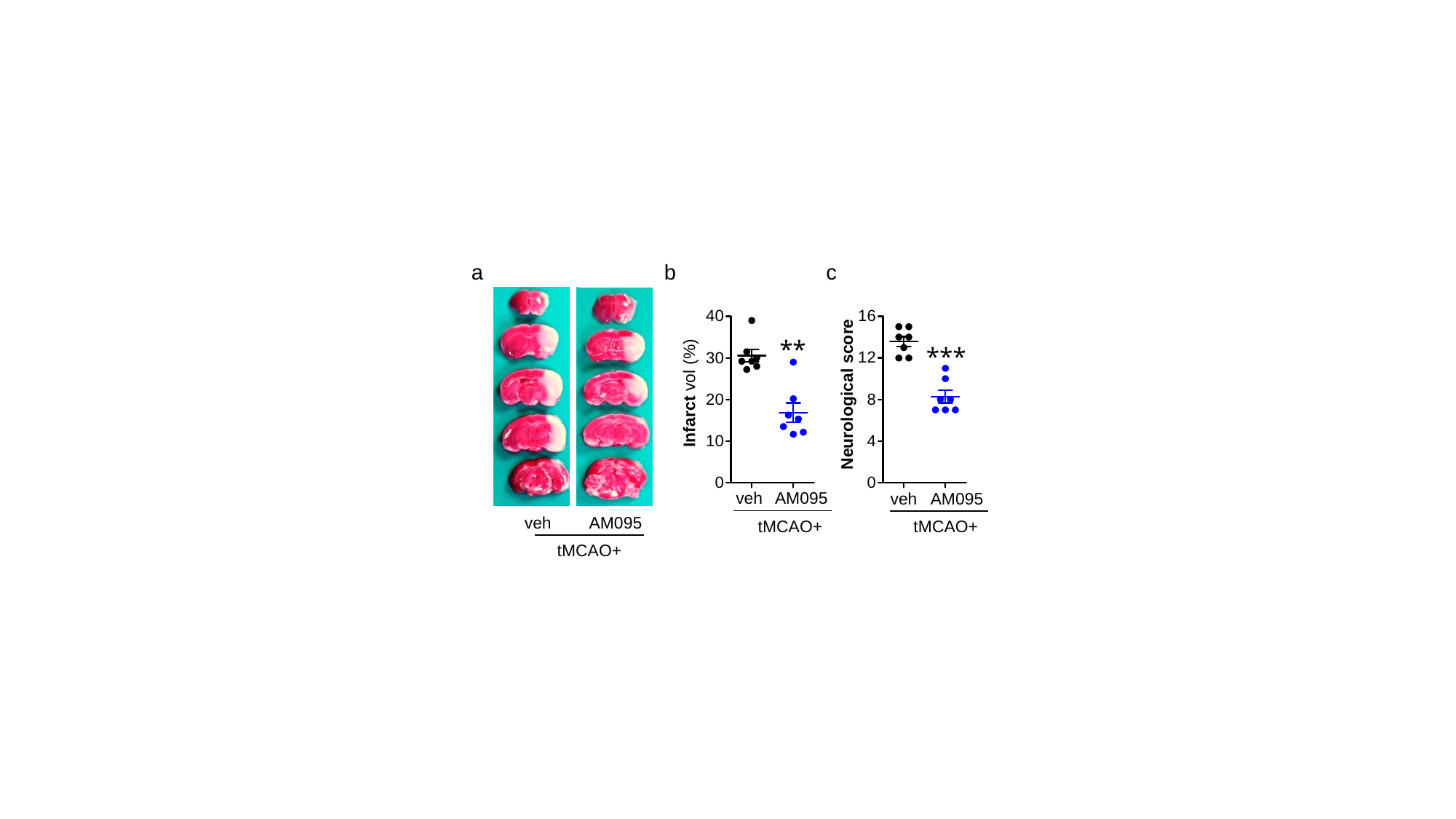

a
b
c
**
***
Infarct vol (%)
Neurological score
veh
AM095
veh
AM095
veh
AM095
tMCAO+
tMCAO+
tMCAO+

Supplement: Supplementary file 3 — Figure S3. Infection with LPA1 shRNA lentivirus causes significant LPA1 knockdown in normal mice brains. Mice were infected with lentivirus for LPA1 shRNA (shLPA1) or non-target control shRNA (shNC) through intracerebroventricular injection of lentivirus particles. Mice brains were then obtained at 7 days later for total RNA extraction. Changes in mRNA expression levels of LPA1 were determined through qRT-PCR analysis. n = 5 mice per group. *p < 0.05 versus mice infected with non-target control shRNA (shNC). (PPTX 403 kb) [file 12974_2019_1555_MOESM3_ESM.pptx]

## Slide 1
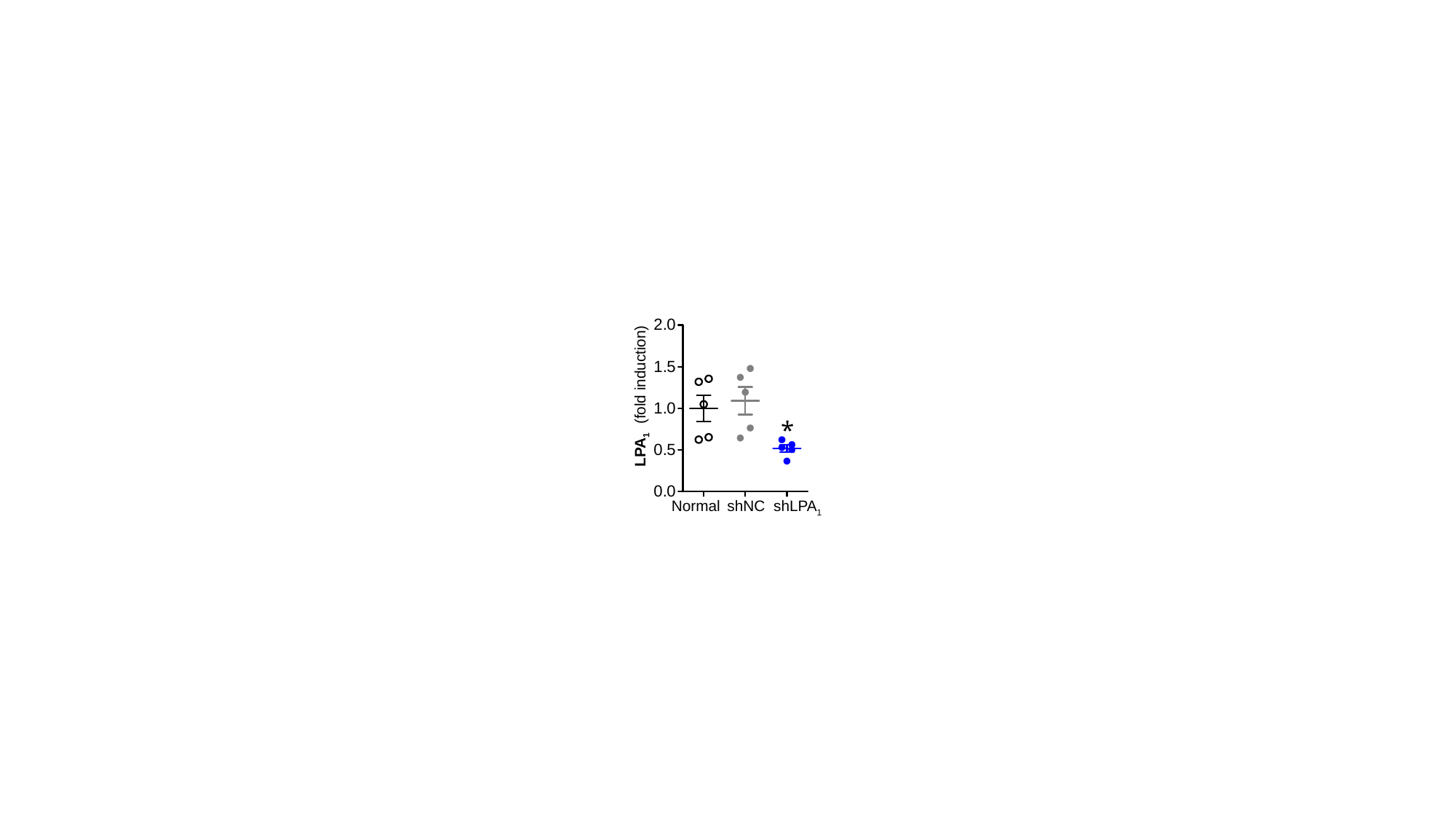

LPA1 (fold induction)
*
Normal
shNC
shLPA1

Supplement: Supplementary file 4 — Figure S4. LPA1 gene expression is reduced in post-ischemic brain. Mice were challenged with tMCAO. Total RNA was extracted from the ipsilateral brain hemisphere at 1 day after tMCAO challenge, and mRNA expression levels of LPA1 were determined using qRT-PCR. Changes in expression levels of LPA1 are shown. n = 5 mice per group. *p < 0.05 versus sham group. (PPTX 36 kb) [file 12974_2019_1555_MOESM4_ESM.pptx]

## Slide 1
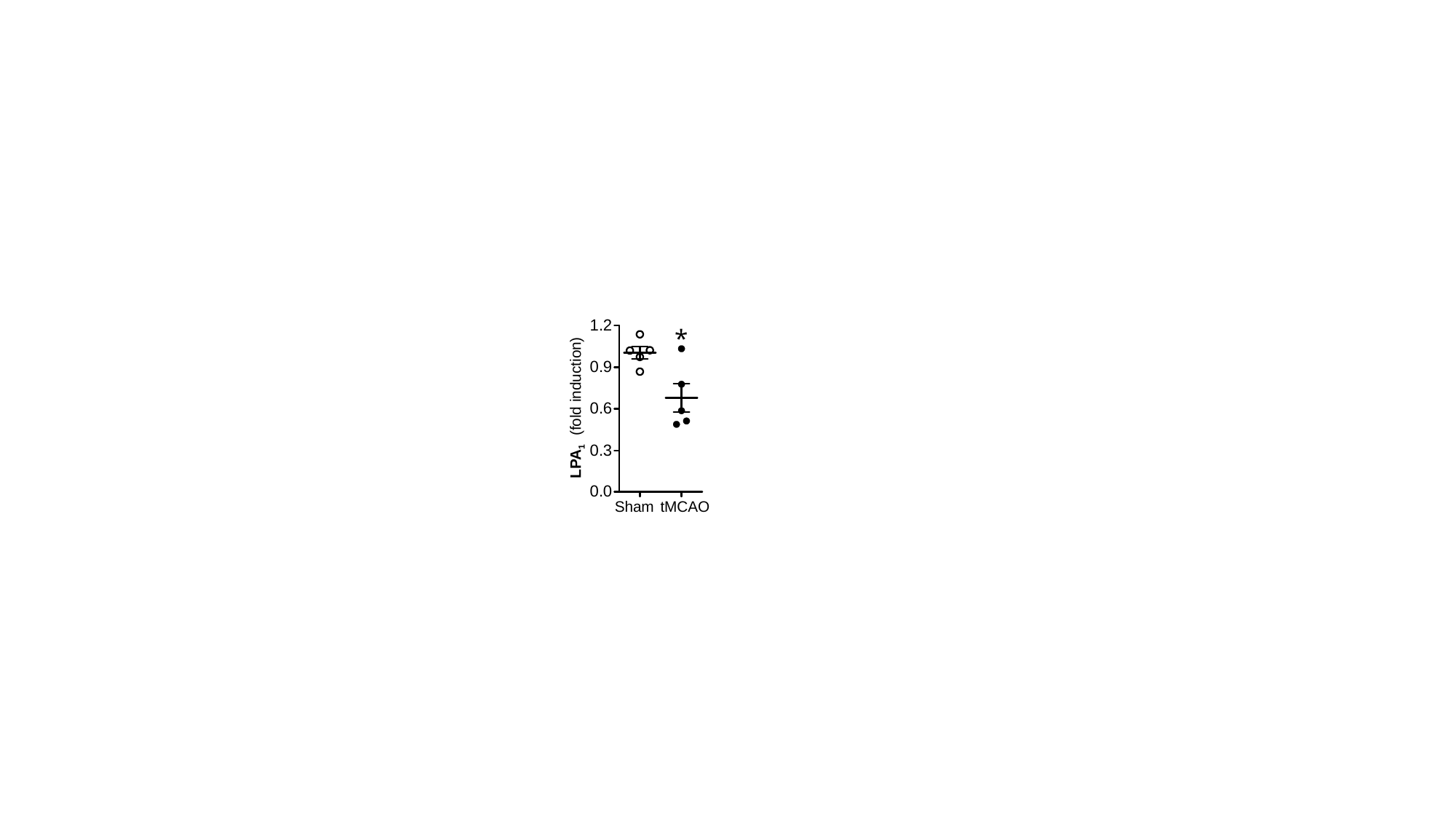

*
LPA1 (fold induction)
Sham
tMCAO

Supplement: Supplementary file 5 — Figure S5. LPA1 knockdown reduces microglial activation in the ischemic brain at 1 day after tMCAO challenge. LPA1 shRNA (shLPA1) and non-target control shRNA (shNC) particles were injected into the ventricle. One week later, mice were challenged with tMCAO. Microglial activation was assessed at 1 day after tMCAO challenge by Iba1 immunohistochemistry. (a) Representative images of Iba1-immunopositive cells in the periischemic (P) and the ischemic core (C) regions. Diagram boxes display the cerebral area where images in middle and bottom panels are acquired. Scale bars, 200 μm (top panels) and 50 μm (middle and bottom panels). Open arrowheads indicate ramified microglia and closed arrowheads indicate amoeboid microglia in the ischemic core region. (b) Quantification of the number of Iba1-immunopositive cells in both regions. (c) Quantification of soma size of Iba1-immunopositive cells in both regions. (d) Quantification of the number of morphologically transferred microglial cells in the ischemic core region (ramified microglia to amoeboid microglia transformation). n = 5 mice per group. ***p < 0.001 versus sham. #p < 0.05, ##p < 0.01, and ###p < 0.001 non-target control lentivirus injected tMCAO mice (tMCAO+shNC). (PPTX 35 kb) [file 12974_2019_1555_MOESM5_ESM.pptx]
