# Supplementary material for: Lysophosphatidic acid receptor 1 (LPA1) plays critical roles in microglial activation and brain damage after transient focal cerebral ischemia
Source: J Neuroinflammation. 2019 Aug 20;16:170. doi: 10.1186/s12974-019-1555-8 (PMC6701099; doi:10.1186/s12974-019-1555-8)
Supplement: Supplementary file 6 — Figure S6. LPA1 knockdown reduces microglial activation in the ischemic brain at 3 days after tMCAO challenge. LPA1 shRNA (shLPA1) and non-target control shRNA (shNC) particles were injected into the ventricle. One week later, mice were challenged with tMCAO. Microglial activation was assessed at 3 days after tMCAO challenge by Iba1 immunohistochemistry. (a) Representative images of Iba1-immunopositive cells in the periischemic (P) and the ischemic core (C) regions. Diagram boxes display the cerebral area where images in middle and bottom panels are acquired. Scale bars, 200 μm (top panels) and 50 μm (middle and bottom panels). Open arrowheads indicate ramified microglia and closed arrowheads indicate amoeboid microglia in the ischemic core region. (b) Quantification of the number of Iba1-immunopositive cells in both regions. (c) Quantification of soma size of Iba1-immunopositive cells in both regions. (d) Quantification of the number of morphologically transferred microglial cells in the ischemic core region (ramified microglia to amoeboid microglia transformation). n = 5 mice per group. **p < 0.01 and ***p < 0.001 versus sham. #p < 0.05, ##p < 0.01, and ###p < 0.001 versus non-target control lentivirus injected tMCAO mice (tMCAO+shNC). (PPTX 7448 kb) [file 12974_2019_1555_MOESM6_ESM.pptx]

## Slide 1
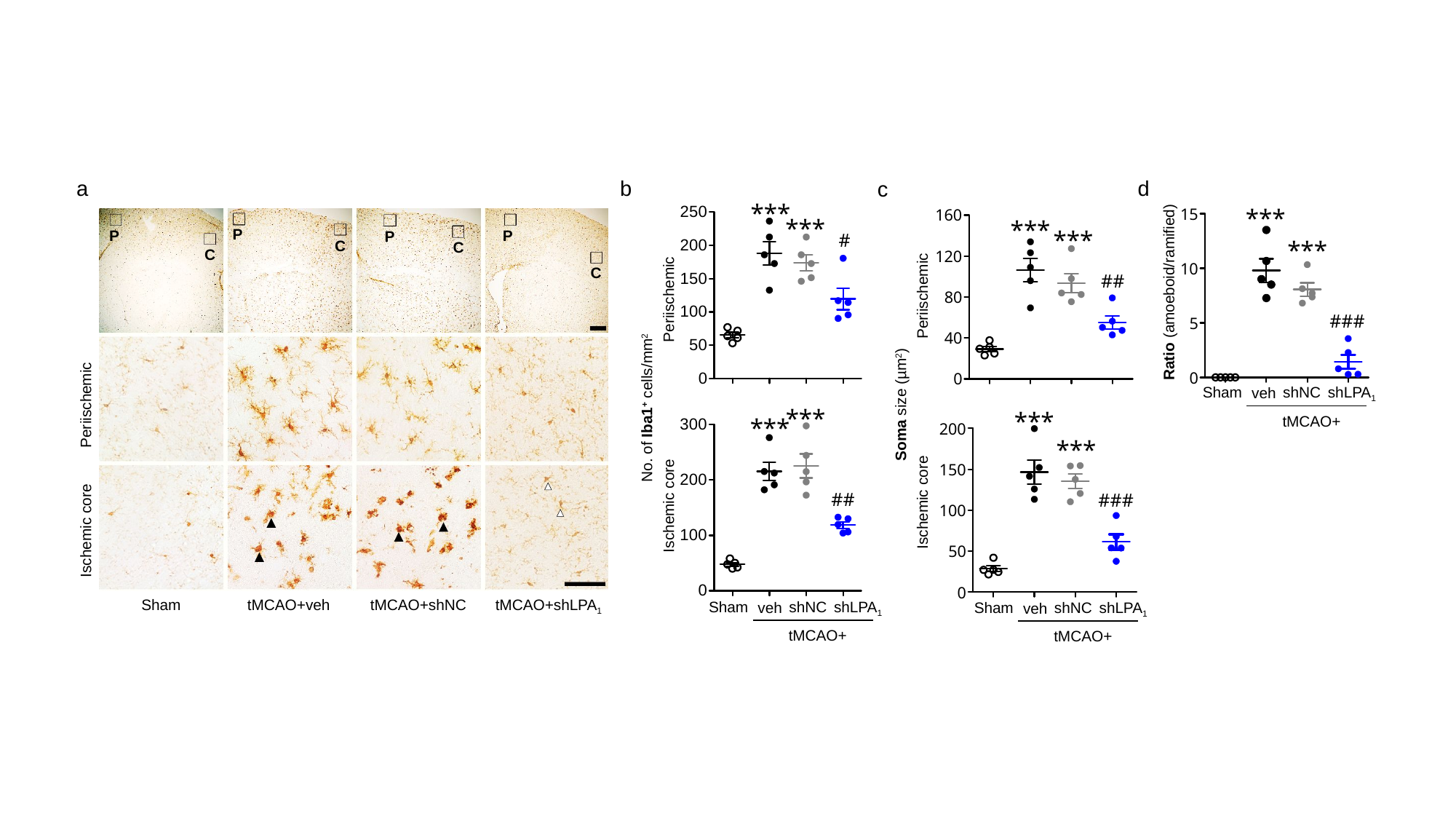

a
b
d
c
***
***
***
***
***
P
P
P
P
#
***
C
C
C
C
##
Ratio (amoeboid/ramified)
Periischemic
Periischemic
###
Sham
shNC
shLPA1
veh
Soma size (µm2)
Periischemic
No. of Iba1+ cells/mm2
***
***
tMCAO+
***
***
##
###
Ischemic core
Ischemic core
Ischemic core
Sham
tMCAO+veh
tMCAO+shNC
tMCAO+shLPA1
Sham
shNC
shLPA1
Sham
shNC
shLPA1
veh
veh
tMCAO+
tMCAO+
