# Supplementary material for: Lysophosphatidic acid receptor 1 (LPA1) plays critical roles in microglial activation and brain damage after transient focal cerebral ischemia
Source: J Neuroinflammation. 2019 Aug 20;16:170. doi: 10.1186/s12974-019-1555-8 (PMC6701099; doi:10.1186/s12974-019-1555-8)
Supplement: Supplementary file 7 — Figure S7. LPA1 knockdown reduces microglial proliferation in the ischemic brain at 3 days after tMCAO challenge. LPA1 shRNA (shLPA1) and non-target control shRNA (shNC) particles were injected into the ventricle. One week later, mice were challenged with tMCAO. BrdU (50 mg/kg in PBS) was administered twice daily at 12 h interval on days 2 and 3 after tMCAO challenge. Microglial proliferation was assessed at 3 days after tMCAO challenge by double immunofluorescence labeling against BrdU and Iba1. (a) Representative images of Iba1/BrdU-double immunopositive cells in the marginal zone (area between the periischemic and the ischemic core regions) of the ischemic brain. Scale bar, 50 μm. (b) Quantification of the number of Iba1/BrdU-double immunopositive cells. n = 5 mice per group. ***p < 0.001 versus sham. #p < 0.05 versus non-target control lentivirus injected tMCAO mice (tMCAO+shNC). (PPTX 2734 kb) [file 12974_2019_1555_MOESM7_ESM.pptx]

## Slide 1
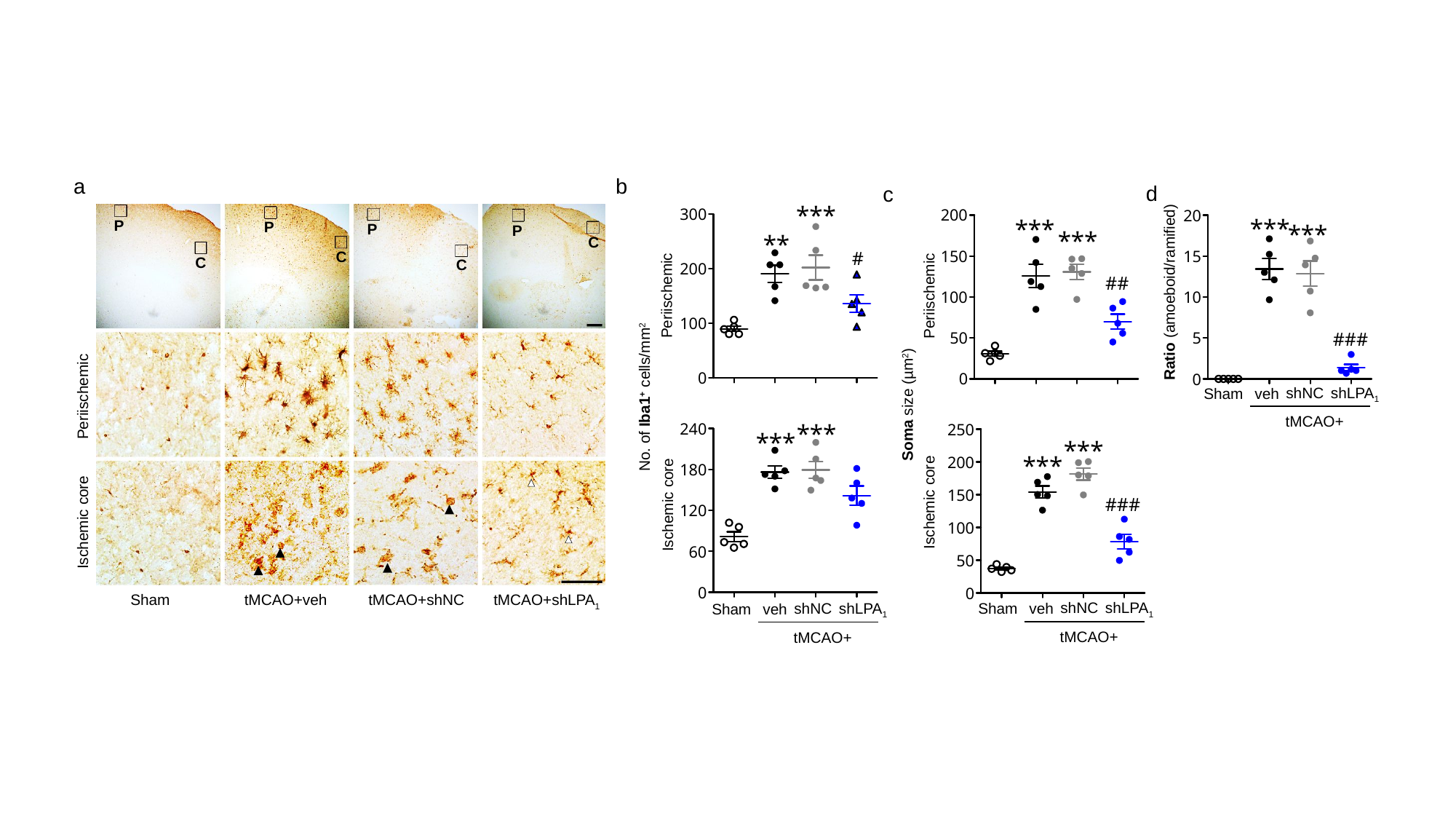

a
b
d
c
***
***
***
P
P
***
P
P
***
**
C
#
C
C
C
##
Ratio (amoeboid/ramified)
Periischemic
Periischemic
###
shNC
shLPA1
Sham
veh
Periischemic
No. of Iba1+ cells/mm2
Soma size (µm2)
tMCAO+
***
***
***
***
###
Ischemic core
Ischemic core
Ischemic core
Sham
tMCAO+veh
tMCAO+shNC
tMCAO+shLPA1
shNC
shLPA1
shNC
shLPA1
Sham
veh
Sham
veh
tMCAO+
tMCAO+
