# Supplementary material for: Lysophosphatidic acid receptor 1 (LPA1) plays critical roles in microglial activation and brain damage after transient focal cerebral ischemia
Source: J Neuroinflammation. 2019 Aug 20;16:170. doi: 10.1186/s12974-019-1555-8 (PMC6701099; doi:10.1186/s12974-019-1555-8)
Supplement: Supplementary file 8 — Figure S8. LPA1 antagonism reduces activation and proliferation of astrocytes in the ischemic brain. Mice were challenged with tMCAO. AM095 (30 mg/kg, p.o.) was administered immediately after reperfusion. Activation of astrocytes was assessed at 1 day and 3 days after tMCAO challenge by GFAP immunohistochemistry. (a) Representative images of GFAP-immunopositive cells in the penumbra area of the corpus callosum. Scale bar, 50 μm. (b, c) Quantification of the number of GFAP-immunopositive cells at 1 day (b) and 3 days (c) after tMCAO challenge. (d, e) Proliferation of astrocytes was assessed at 3 days after tMCAO challenge by double immunofluorescence labeling against BrdU and GFAP. (d) Representative images of GFAP/BrdU-double immunopositive cells in the marginal zone (area between the periischemic and the ischemic core regions) of the ischemic brain. Scale bar, 50 μm. (e) Quantification of the number of GFAP/BrdU-double immunopositive cells. n = 5 (sham), 5 (tMCAO+veh), and 4 (tMCAO+AM095). ***p < 0.001 versus sham. ##p < 0.01 and ###p < 0.001 versus vehicle-administered tMCAO mice (tMCAO+veh). (PPTX 1610 kb) [file 12974_2019_1555_MOESM8_ESM.pptx]

## Slide 1
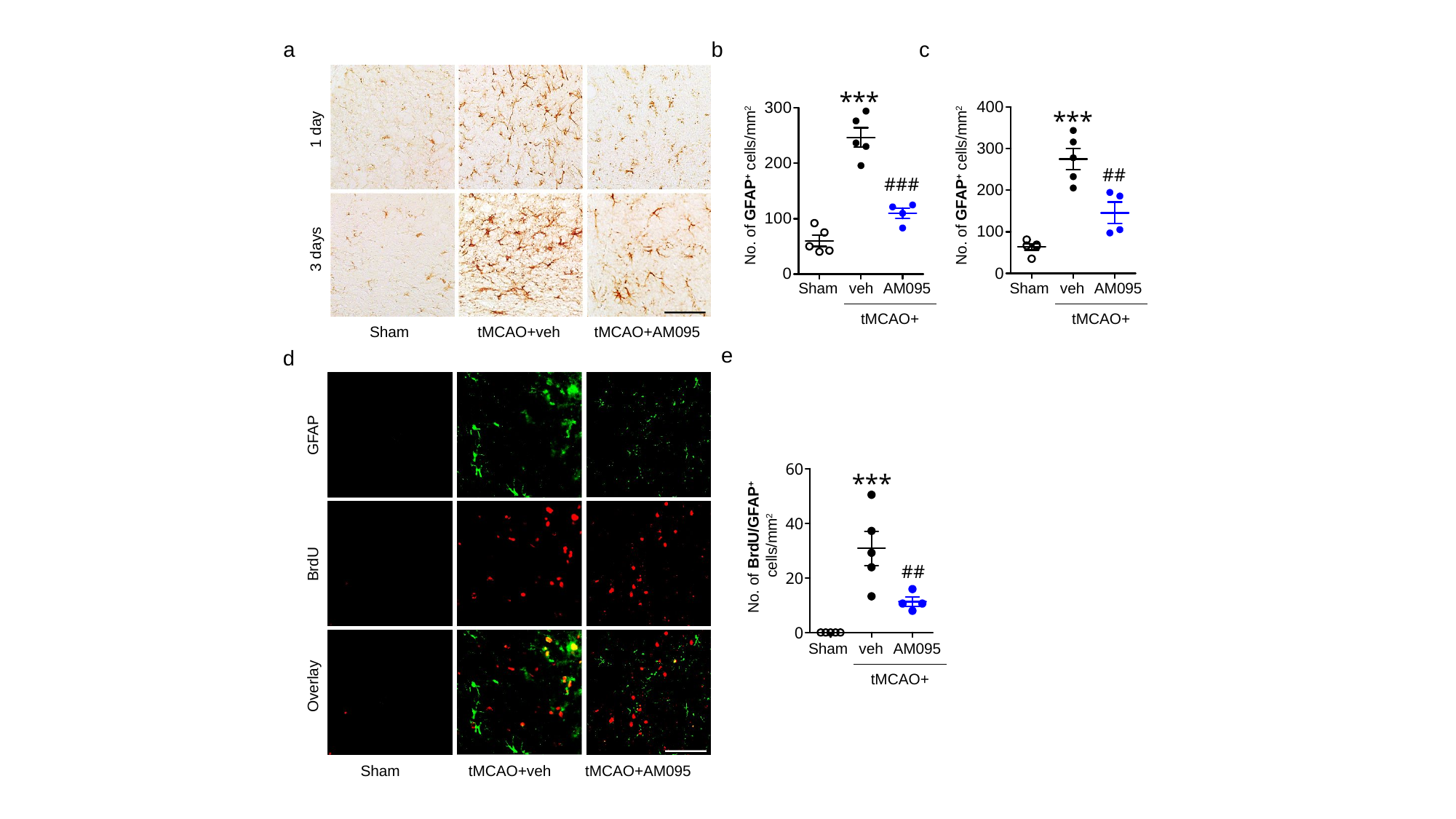

a
b
c
***
***
1 day
##
###
No. of GFAP+ cells/mm2
No. of GFAP+ cells/mm2
3 days
Sham
veh
AM095
Sham
veh
AM095
tMCAO+
tMCAO+
Sham
tMCAO+veh
tMCAO+AM095
e
d
GFAP
***
No. of BrdU/GFAP+
cells/mm2
BrdU
##
Sham
veh
AM095
tMCAO+
Overlay
Sham
tMCAO+veh
tMCAO+AM095
