# Supplementary material for: Lysophosphatidic acid receptor 1 (LPA1) plays critical roles in microglial activation and brain damage after transient focal cerebral ischemia
Source: J Neuroinflammation. 2019 Aug 20;16:170. doi: 10.1186/s12974-019-1555-8 (PMC6701099; doi:10.1186/s12974-019-1555-8)
Supplement: Supplementary file 9 — Figure S9. LPA1 antagonism alters expression levels of few anti-inflammatory cytokines in the ischemic brain after tMCAO challenge. Mice were challenged with tMCAO. AM095 (30 mg/kg, p.o.) was administered immediately after reperfusion. Total RNA was extracted from the ipsilateral brain hemisphere at 1 day and 3 days after tMCAO challenge, and mRNA expression levels of anti-inflammatory cytokines were determined by qRT-PCR analysis. Changes in expression levels of anti-inflammatory cytokines at 1 day (a-c) and at 3 days (d-f) are shown. n = 5 mice per group. ***p < 0.001 versus sham. ##p < 0.01 versus vehicle-administered tMCAO mice (tMCAO+veh). (PPTX 54 kb) [file 12974_2019_1555_MOESM9_ESM.pptx]

## Slide 1
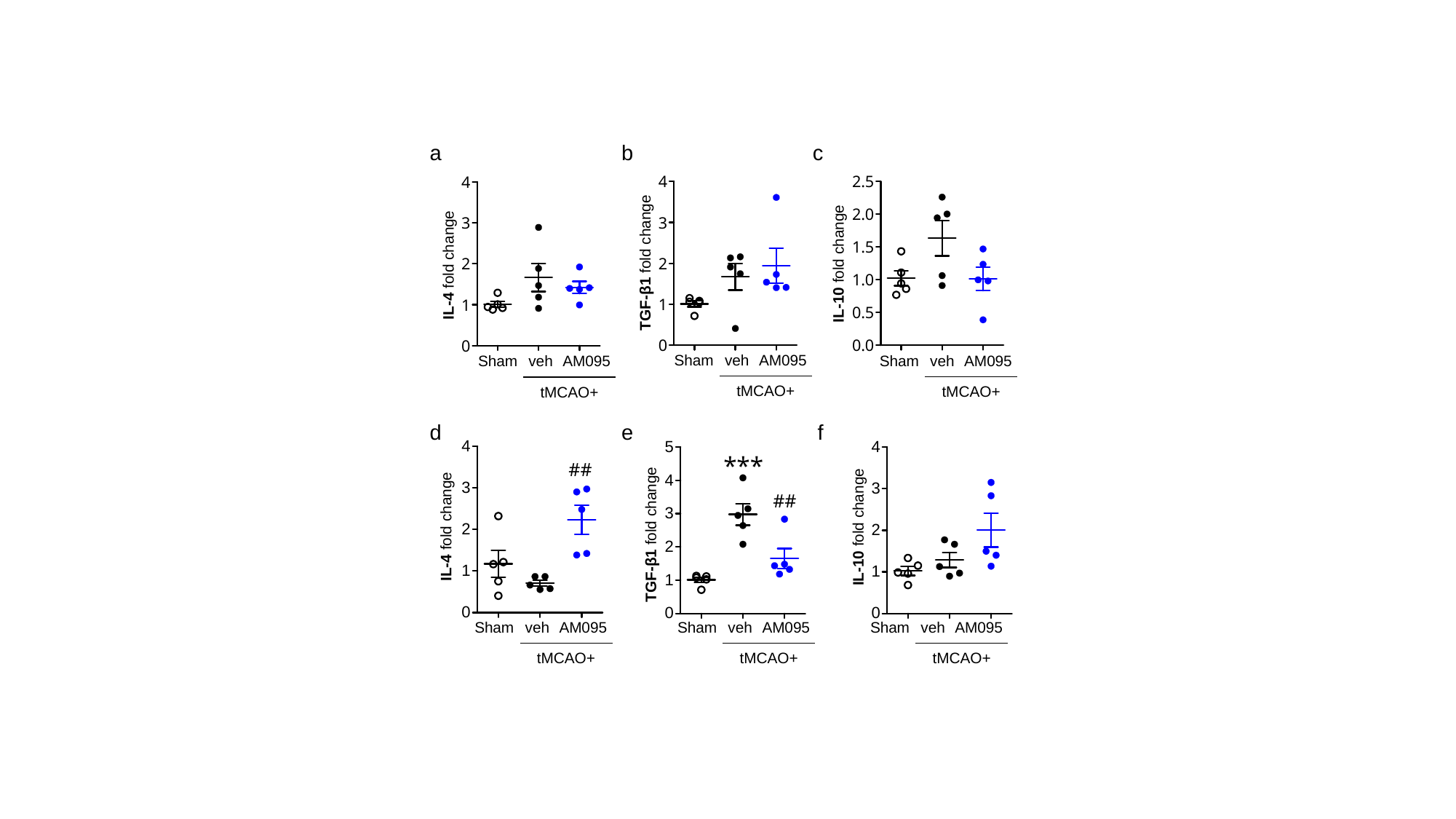

a
b
c
TGF-β1 fold change
IL-10 fold change
IL-4 fold change
Sham
veh
AM095
Sham
veh
AM095
Sham
veh
AM095
tMCAO+
tMCAO+
tMCAO+
f
d
e
***
##
##
IL-4 fold change
IL-10 fold change
TGF-β1 fold change
Sham
veh
AM095
Sham
veh
AM095
Sham
veh
AM095
tMCAO+
tMCAO+
tMCAO+
