# Supplementary material for: Lysophosphatidic acid receptor 1 (LPA1) plays critical roles in microglial activation and brain damage after transient focal cerebral ischemia
Source: J Neuroinflammation. 2019 Aug 20;16:170. doi: 10.1186/s12974-019-1555-8 (PMC6701099; doi:10.1186/s12974-019-1555-8)
Supplement: Supplementary file 10 — Table S1. Primer sets used for qRT-PCR analysis. (PDF 293 kb) [file 12974_2019_1555_MOESM10_ESM.pdf]

Table S1. Primer sets used for qRT-PCR analysis

Target	Direction	Sequence	Gene Accession #
β-actin	Forward	5'-AGCCTTCCTTCTTGGGTATG-3'	NM_007393
	Reverse	5'-CTTCTGCATCCTGTCAGCAA-3'	
TNF-α	Forward	5'-CATCTTCTCAAAATTCGAGTGACAA-3'	NM_013693
	Reverse	5'-TGGGAGTAGACAAGGTACAACCC-3'	
IL-1β	Forward	5'-CAACCAACAAGTGATATTCTCCATG-3'	NM_008361
	Reverse	5'-GATCCACACTCTCCAGCTGCA-3'	
IL-6	Forward	5'-GAGGATACCACTCCCAACAGACC-3'	NM_031168
	Reverse	5'-AAGTGCATCATCGTTGTTCATACA-3'	
LPA_1	Forward	5'-TCTTCTGGGCCATTTTCAAC-3'	NM_010336.2
	Reverse	5'-TGCCTGAAGGTGGCGCTCAT-3'	
IL-10	Forward	5'-TGGCCTTGTAGACACCTTGG-3'	NM_010548
	Reverse	5'-AGCTGAAGACCCTCAGGATG-3'	
IL-4	Forward	5'-GTCATCCTGCTCTTCTTTCTCG-3'	NM_021283.2
	Reverse	5'-TCTGTGGTGTTCTTCGTTGCT-3'	
TGF-β1	Forward	5'-CAACCCAGGTCCTTCCTAAA-3'	NM_011577
	Reverse	5'-GGAGAGCCCTGGATACCAAC-3'	
